# Supplementary material for: Structural and functional studies of the EGF20-27 region reveal new features of the human Notch receptor important for optimal activation
Source: Structure. 2024 Dec 5;32(12):2325–2336.e5. doi: 10.1016/j.str.2024.10.012 (PMC12979247; doi:10.1016/j.str.2024.10.012)
Supplement: Document S1. Figures S1–S8 and Table S1 [file mmc1.pdf]

Structure, Volume *32*

Supplemental Information

Structural and functional studies of the EGF20-27 region reveal new features of the human Notch receptor important for optimal activation

Zhihan Bo, Thomas Rowntree, Steven Johnson, Hilman Nurmahdi, Richard J. Suckling, Johan Hill, Boguslawa Korona, Philip C. Weisshuhn, Devon Sheppard, Yao Meng, Shaoyan Liang, Edward D. Lowe, Susan M. Lea, Christina Redfield, and Penny A. Handford

Supplemental Information

Structural and functional studies of the EGF20-27 region reveal new features of the Notch receptor important for optimal activation.

Zhihan Bo[1‡], Thomas Rowntree[1‡], Steven Johnson[2#‡], Hilman Nurmahdi[1], Richard Suckling[2], Johan Hill[1], Bogusia Korona[1], Philip C. Weisshuhn[1], Devon Sheppard[2], Yao Meng[1], Shaoyan Liang[1], Edward D. Lowe[1], Susan M. Lea[2#*], Christina Redfield[1,*] & Penny A. Handford[1, *]

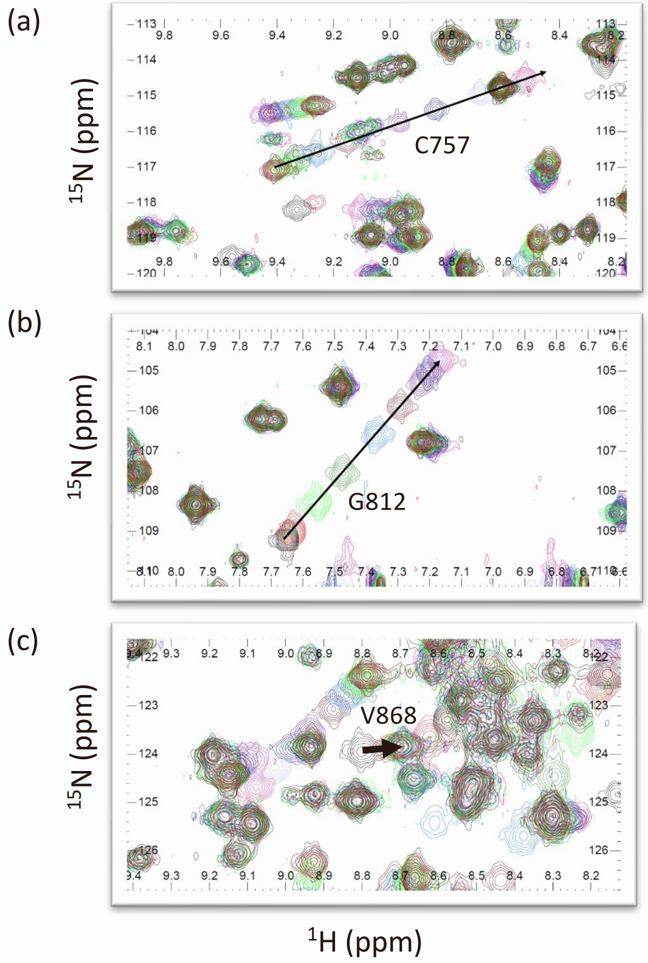

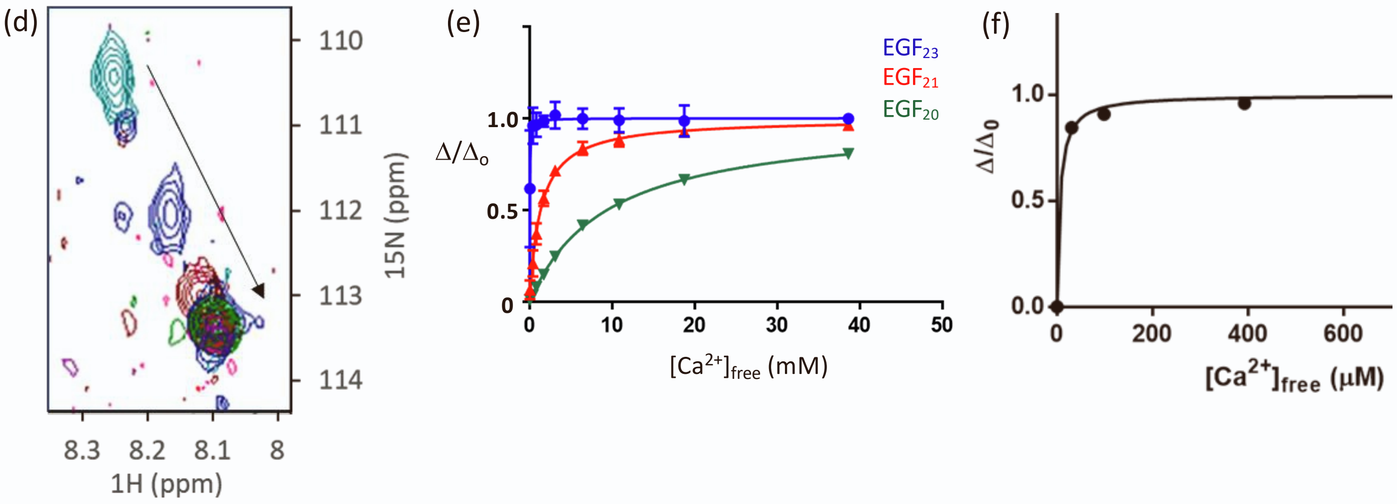

Figure S1 Determination of Ca^{2+} K_D values for Ca^{2+}-binding domains of hNotch-1 (EGF20, EGF21, EGF23 and EGF24) from ^{1}H-^{15}N HSQC spectra collected at 500 MHz for EGF20-23 and EGF23-24. Related to Figure 1.

(a)-(c) Overlays of zoomed in regions of HSQC spectra of EGF20-23 collected at 298K for Ca^{2+} concentrations of 0, 0.5, 1.0, 1.5, 2.5, 4.0, 7.5, 12.0, 20.0 and 40.0 mM showing Ca^{2+} dependent chemical shift changes. (a) The arrow indicates how the C757 peak in EGF20 shifts with increasing [Ca^{2+}]. (b) The arrow indicates how the G812 peak in EGF21 shifts with increasing [Ca^{2+}]. (c) The arrow indicates how the V868 peak in EGF23 shifts with increasing [Ca^{2+}]. (d) Overlay of zoomed in regions of HSQC spectra of EGF23-24 collected at 298K for Ca^{2+} concentrations of 0, 0.1, 0.2 and 0.6 mM showing Ca^{2+} dependent chemical shift changes. The arrow indicates how a peak arising from a glycine residue in EGF24 shifts with increasing [Ca^{2+}]. (e)-(f) Fractional changes (Δ/Δ_o) in the combined HN/^{15}N chemical shifts for residues in EGF20, EGF21 and EGF23 (e) and in EGF24 (f) are plotted against the concentration of free Ca^{2+} to yield K_D values of 9mM, 1.3mM, 23μM and <25μM for EGF20, EGF21, EGF23 and EGF24, respectively. Chemical shift changes were fitted to the equation $\Delta/\Delta_o = [Ca^{2+}]_{free}/([Ca^{2+}]_{free} + K_D)$, where Δ is the observed chemical shift change at each value of $[Ca^{2+}]_{free}$ and Δ_o is the maximum observed chemical shift change. Error bars shown for Δ/Δ_o represent estimated errors involved in measuring chemical shift values.

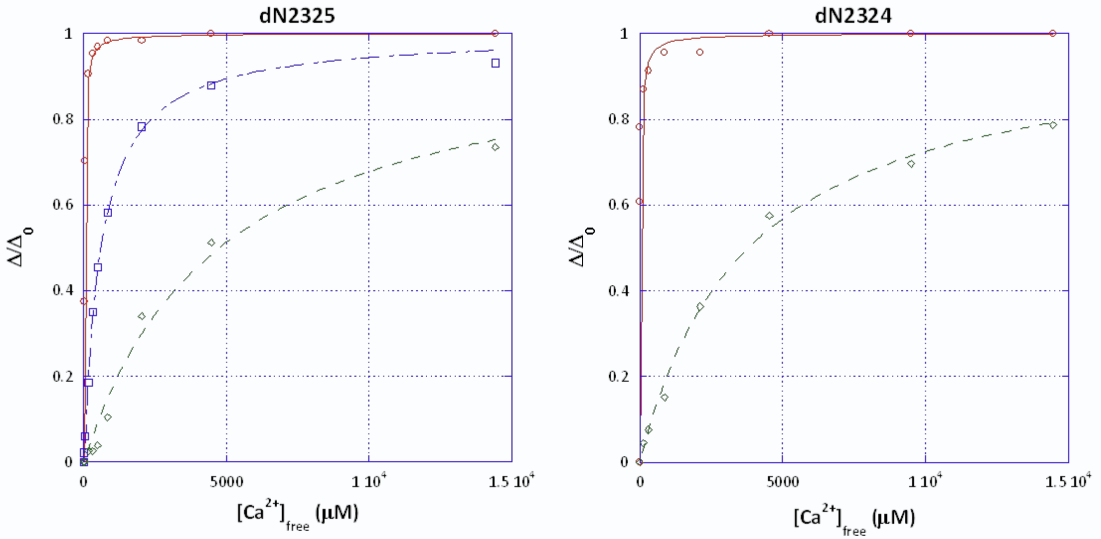

Figure S2. Quantification of Ca^{2+}-binding sites in Drosophila Notch (dN) EGF23-25, EGF23-24 by 2D-NOESY. Related to Figure 1.

Plots of Ca^{2+}-dependent chemical shift changes in EGF23, 24, and 25 are coloured green, red and blue respectively. K_D values of each domain in dN EGF23-25 were ~5mM, ~20μM and~600μM respectively. K_D values of EGF23 and 24 in dN EGF23-24 were ~4mM and ~20μM respectively.

Figure S3. Quantification of Ca^{2+}-binding sites in Drosophila Notch (dN) EGF23-25, EGF23-24, and Abruptex variants EGF23-25_948 (D948V in EGF24), and EGF23-25_986 (N986I in EGF25) by 2D-NOESY. Related to Figure 1.

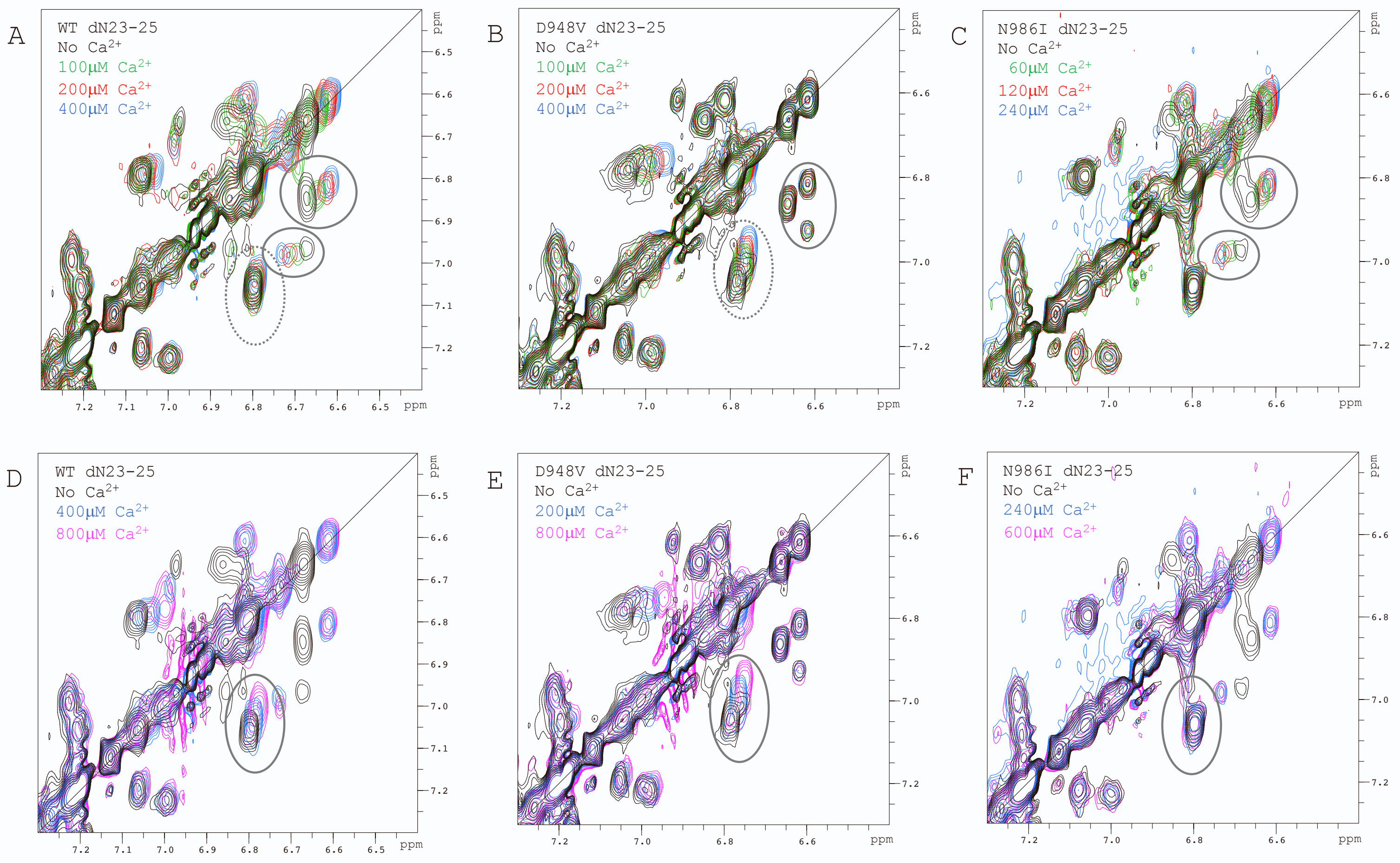

Figure S3 legend. Quantification of Ca^{2+}-binding sites in Drosophila Notch (dN) EGF23-25, EGF23-24, and Abruptex variants EGF23-25_948 (D948V in EGF24), and EGF23-25_986 (N986I in EGF25) by 2D-NOESY. Related to Figure 1.

2D ^{1}H-^{1}H NOESY spectra for WT EGF23-25 and the two variants at various [Ca^{2+}] were used to characterise Ca^{2+} binding in EGF24 and EGF25. In A, peaks from aromatic residues assigned to EGF24 (solid gray ovals) shift at Ca^{2+} concentrations between 0 and 400 μM indicating a relatively high affinity binding site. The concentration of the WT EGF23-25 was 280μM. These peaks were assigned to EGF24 by comparison with spectra for EGF23-24. In B, these peaks do not move indicating that the D948V substitution in EGF24 greatly reduces the affinity for Ca^{2+} in EGF24. In C, these peaks shift in a manner similar to that in WT EGF23-25 indicating that the N986I substitution in EGF25 does not affect the affinity for Ca^{2+} in EGF24. In D, a peak from an aromatic residue assigned to EGF25 (solid gray oval) shifts at Ca^{2+} concentrations between 0 and 800 μM. This peak is highlighted in A by the dotted oval. Comparison of A and D shows that this peak only shifts at higher Ca^{2+} concentrations, indicating a weaker affinity binding site in EGF25 compared to EGF24. This peak was assigned to EGF25 by comparison with spectra for EGF23-24, where it is absent. In E, this peak shifts in a manner similar to that seen in D, indicating that the D948V substitution in EGF24 does not affect the affinity for Ca^{2+} in EGF25. In F, this peak does not move indicating that the N986I substitution in EGF25 greatly reduces the affinity for Ca^{2+} in EGF25.

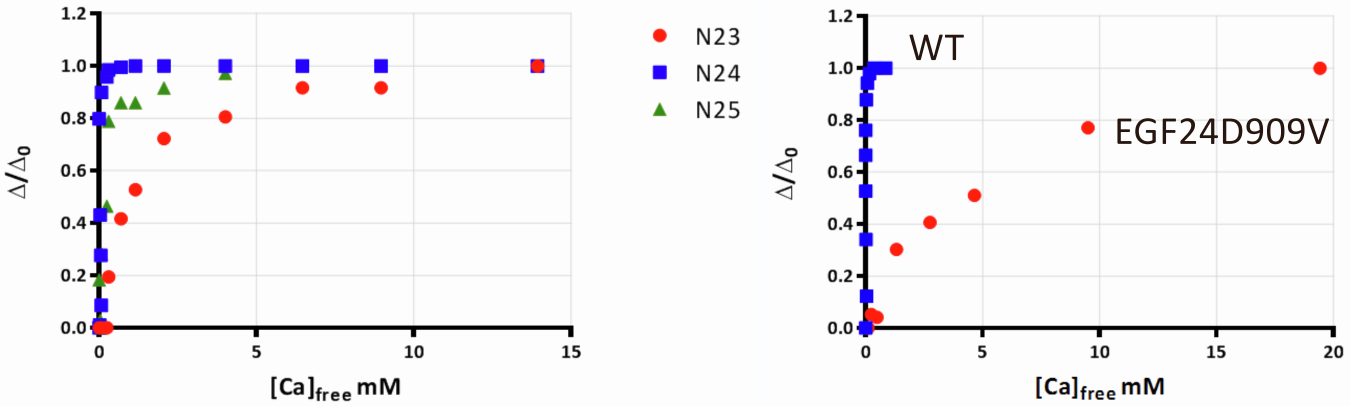

Figure S4. Analysis of homologous Abruptex missense mutation D909V in hNotch-1 EGF24. Related to Figure 1.

hNotch-1 23-25 has 3 calcium-binding sites. D909V reduces the affinity of EGF24 site by 50-100x

Figure S5 SAXS data for hNotch-1 EGF23-27 compared to EGF20-27 . Related to Figure 4.

A scaled, merged and averaged X-ray scattering curve collected with purified hNotch-1 EGF23-27 at 2.5 mg/ml in 5 mM Tris, pH 7.5, 15 mM $CaCl_2$. Data are shown compared to EGF20-27. The P(r) distribution shows the expected difference in length for the two fragments comprising 5 and 8 EGF domains respectively.

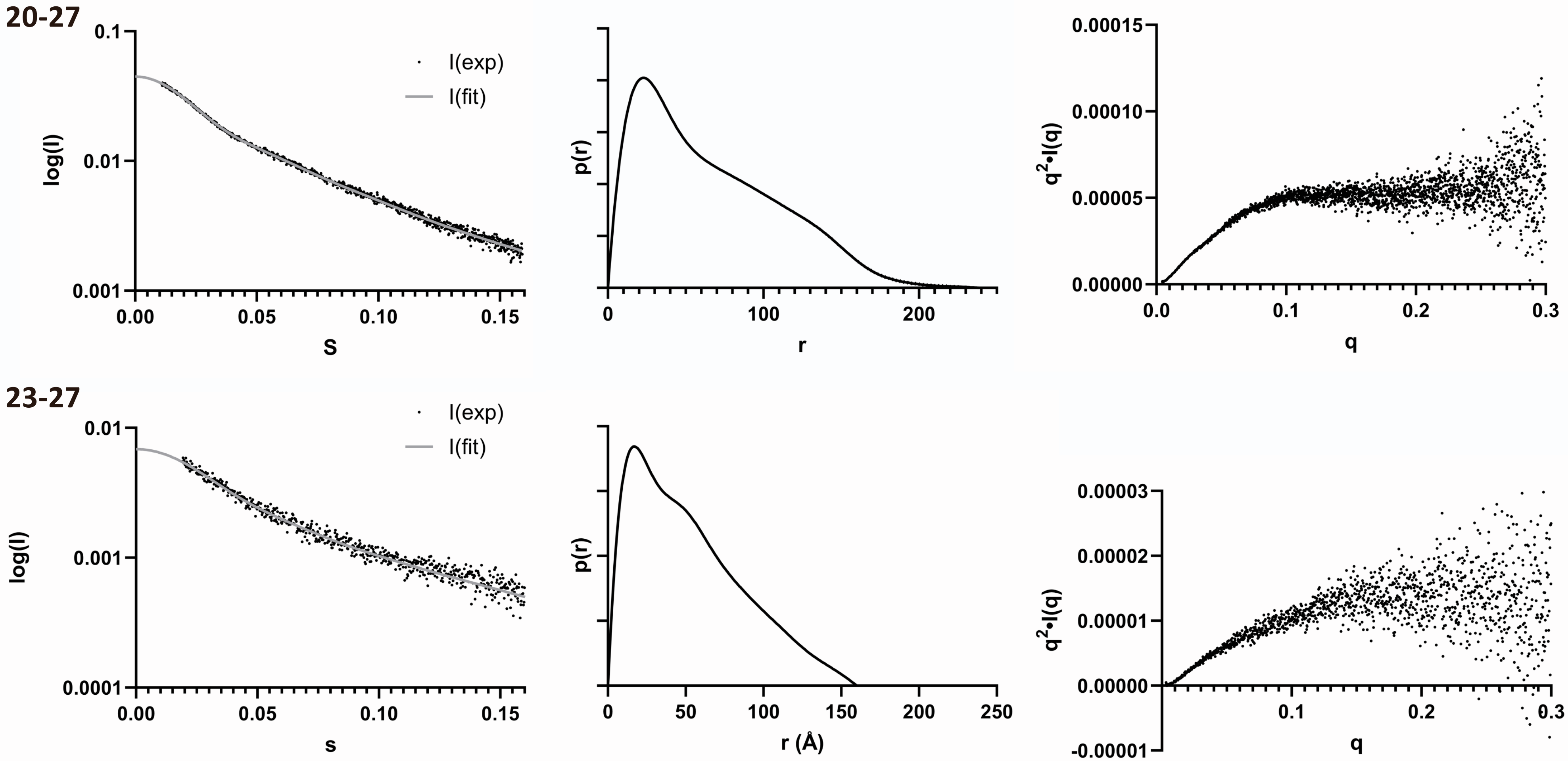

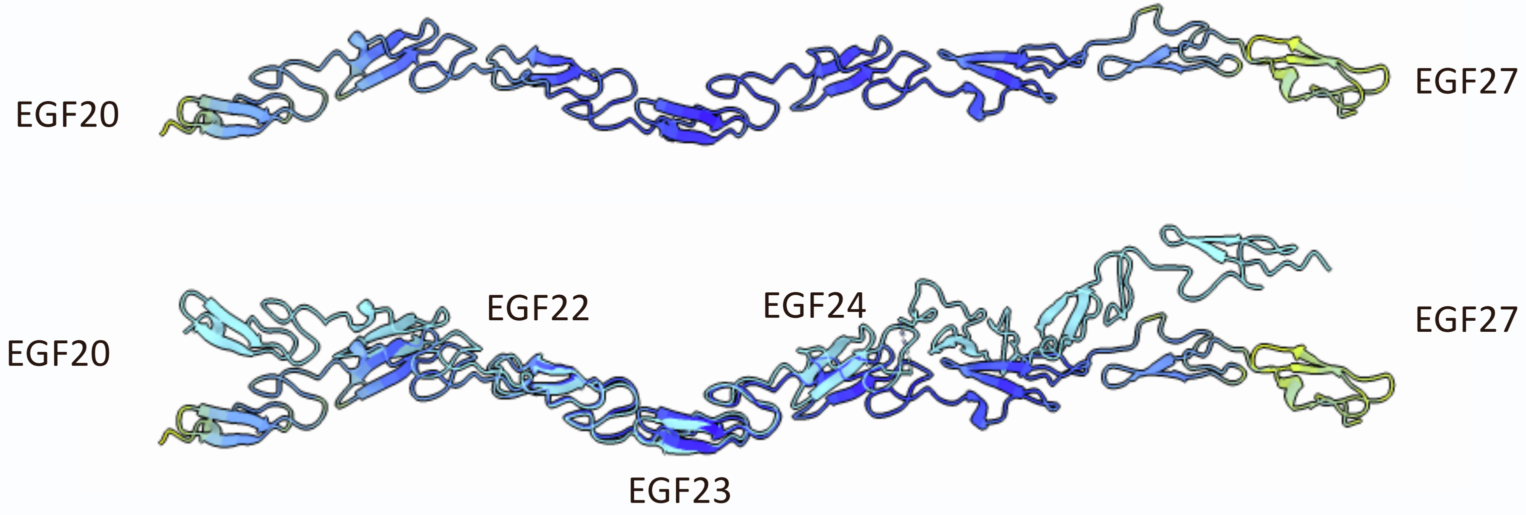

Figure S6. Comparison of AlphaFold2 model for EGF20-27 with the model derived from the SAXS data. Related to Figure 4.

Top: Cartoon representation of the highest ranked AlphaFold2 model generated for EGF20-27. Colouring is based on the pLDDT confidence score (high confidence in dark blue, low confidence in yellow).

Bottom: The AlphaFold2 model is aligned with the model generated by SREFLEX using the SAXS data.

The AlphaFold2 colouring is the same as in the top figure while the SREFLEX structure is shown in light blue.

The two models overlay well for domains EGF22-24 but deviate at both the N- and C-termini. Although both models show an elongated structure, the AlphaFold2 model appears to be slightly more extended. The SREFLEX model gives better agreement with the SAXS data ($\chi 2$ = 1.96) than the AlphaFold model ($\chi 2$ = 3.35), as assessed by CRYSOL.

Figure S7. Luciferase reporter assays of Notch activity. Related to Figure 5.

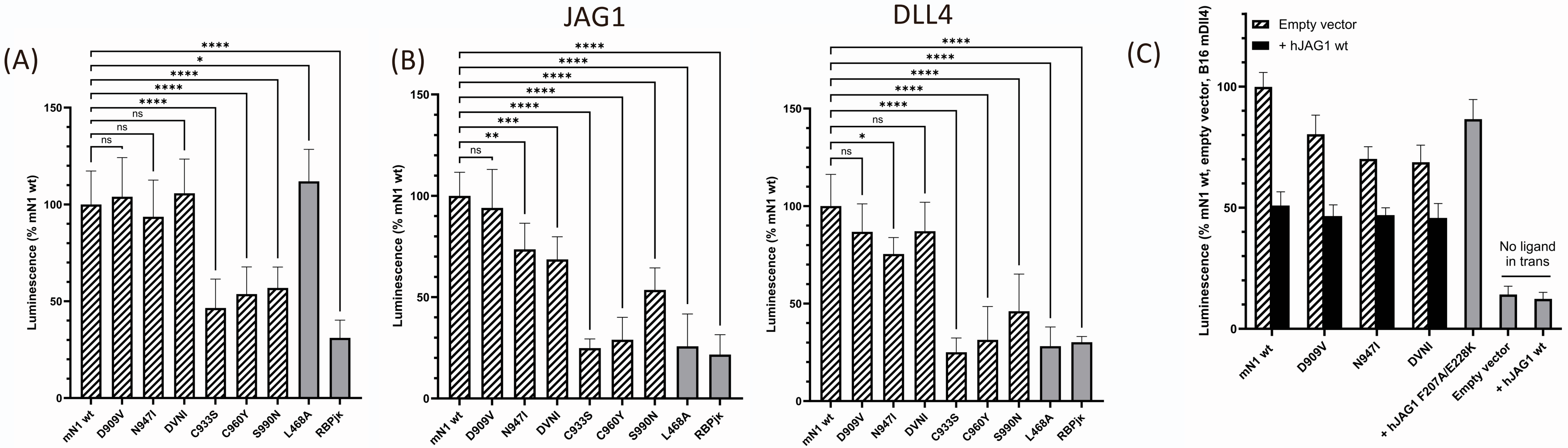

(A) Ligand-independent activation: Graphs show combined results of four independent plates, each with five repeat wells. The signal in each case is shown relative to Notch-1 wt (mN1 wt). Data presented as mean ± SD. Statistical significance was determined by ordinary one-way ANOVA and Dunnett post-hoc test (ns= no significance, * = P < 0.05, ** = P < 0.01, *** = P < 0.001, **** = P < 0.0001).

(B) Ligand-dependent transactivation using purified NE3 ligand constructs: Graphs show the combined results of four independent plates, each with five repeat wells. The signal in each case is shown relative to Notch-1 wt (mN1 wt). Data presented as mean ± SD. Statistical significance was determined by Kruskal-Wallis test and Dunn's post hoc test (ns= no significance, * = P < 0.05, ** = P < 0.01, *** = P < 0.001, **** = P < 0.0001).

(C) Cis-inhibition of Notch variants co-cultured with B16 cells expressing mDll4: Graphs show combined results of 4 independent plates, each with five repeat wells. Signal in each case is shown relative to Notch-1 wt (mN1 wt) transfected with empty pcDNA3.1, co-cultured with B16 mDll4 expressing cells. Data presented as mean ± SD.

(D) Direct measurement of cell surface Notch variants by FACS: Data presented as mean ± SD. Statistical significance was determined by Kruskal-Wallis test and Dunn's post hoc test (ns = not significant, * p<0.05, ** p<0.01, *** p<0.001, **** p < 0.0001).

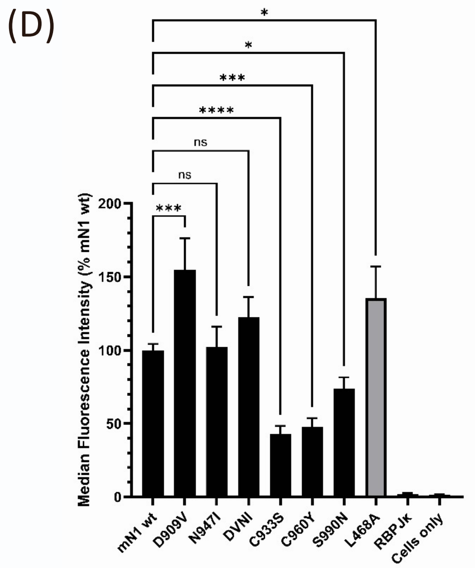

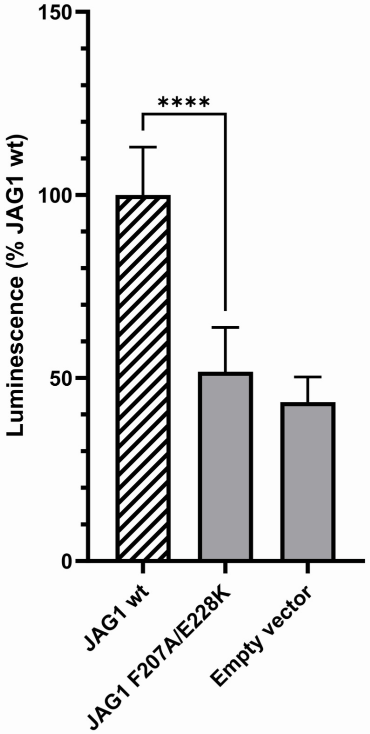

Figure S8. Activity data showing full-length JAG1 variant F207A/E228K has a reduced ability to transactivate WT Notch-1, as well as a reduced ability to cis-inhibit, suggesting the ligand DSL domain -Notch LBR interaction may be common to both modes of regulation. Related to Figure 5.

Luciferase reporter assay shows JAG1 F207A/E228K variant has a reduced ability to trans-activate Notch-1. Notch-1 wt cell line co-cultured with cells transfected with pcDNA3.1 full-length hJAG1 wt, pcDNA3.1 hJAG1F207A/E228K, or empty pcDNA3.1 vector. Graphs show the combined results of four independent plates, each with five repeat wells. The signal in each case is shown relative to JAG1 wt. Data presented as mean $\pm$ SD. Statistical significance was determined by Mann-Whitney test (**** = $P < 0.0001$).

Table S1. Primers used for cloning and mutagenesis in this study. Primers were purchased from Thermo Fisher Scientific. Related to STAR Methods.

Primer	Sequence (5' - 3')
pcDNA5/FRT mN-1 mutagenesis	
For_end_Sbf1	ACACCTGCAGGCAAGATGTTAATGAGTGCAGCCAGAACCC
Rev_end_BamH1	GAAGGATCCCAGGCATAGGCAGGTGGGACTACGTGGG
mouseN1_L468A_F	CAGAATGATGCCACTTGCGCCGACCAGATTGGGGAGTTC
mouseN1_L468A_R	GAACTCCCCAATCTGGTCGGCGCAAGTGGCATCATTCTG
mouseN1_D909V_F	AACTGTGAGAGTGACATCGTGGACTGCCGCCCCAACCCG
mouseN1_D909V_R	CGGGTTGGGGCGGCAGTCCACGATGTCACTCTCACAGTT
mouseN1_C933S_F	CTTCTGCGACAGTCTGCCCGGCTTCCAGGGTG
mouseN1_C933S_R	CCGGGCAGACTGTCGCAGAAGGCTGTGTTGATG
mouseN1_N947I_F	TTCTGTGAGGAGGACATCATTGAATGTGCCAGCAATCCC
mouseN1_N947I_R	GGGATTGCTGGCACATTCAATGATGTCCTCCTCACAGAA
mouseN1_C960Y_F_2	CCAAAATGGTGCCAATTATACTGACTGTGTGGACAGCTACAC
mouseN1_C960Y_R_2	GCTGTCCACACAGTCAGTATAATTGGCACCATTTTGGCAGGG
mouseN1_S990N_F_2	CACCTGACTGTACTGAGAATTCCTGCTTCAATGGTGGTACCTG
mouseN1_S990N_R_2	CCACCATTGAAGCAGGAATTCTCAGTACAGTCAGGTGTGTTG
pQE30 hNotch-1 EGF21-23 cloning	
EGF21EK_F	TAGTAGGGATCCGATGACGATGACAAATCAGCAACCAACATCAACG AGTGTGCGTC
EGF23_R	TAGTAGAAGCTTCTATTAGGTCTCGCAGTTGCGCCCACTG
pEXS2.2 hNotch-1 EGF20-24 cloning	
F4_N110-13	TTAAGAATTCGCCACCATGAAGCTGTGCATC
AbiN1-24R	ATAGGATCCCTCCTCGCAGAAGGTGCCGCG
pEXS2.2 hNotch-1 EGF20-27 cloning	
F4_N110-13	TTAAGAATTCGCCACCATGAAGCTGTGCATC
AbiN1-27R	ATAGGATCCGTTCTGGCAATTCGGACCGGTG
pQE30 hNotch-1 EGF23-27 cloning	
23-27CSCS_F	TAGTAGAGAGATCTATAGAAGGACGATCAGCAGTCGACATCAACGA GTGCG
23-27CSCS_R	CACTGGCCCCAACTGCCAGCTCGAGTAGTAG
pQE30 dNotch EGF23-24 cloning	
Forward	TAGTAGGGATCCATAGAAGGACGATCAGCAGAGGACATAGACGAA TGCTC
Reverse	CTACTAGTCGACCGTCTCGCAGTGCTTGCC
pQE30 dNotch EGF23-25 cloning	
Forward	TAGTAGGGATCCATAGAAGGACGATCAGCAGAGGACATAGACGAA TGCTC
Reverse	CTACTAGTCGACCGTCTGACAATTGATGCCGG
pQE30 dNotch EGF23-24 and EGF23-25 mutagenesis	
dN2325_948_Forward	CATCAATACGGTCGATTGTG
dN2325_948_Reverse	GCGCAATCTCTGCCCTCA
dN2325_986_Forward	GACATCATTGAGTGCTTGAG
dN2325_986_Reverse	CGTCTCGCAGTGCTTGCC
pQE30 hNotch-1 EGF23-24 cloning	

EGF23 Fwd	TAGTAGAGATCTATAGAAGGACGATCAGCAGTCGACATCAACGAGTGCG
EGF24 Rev	TAGTAGCTCGAGCTCACAGAAAGTGCCCCGG
pQE30 hNotch-1 EGF23-24 mutagenesis	
SDM D909V Fwd	GACATCGTCGACTGCCGG
SDM D909V Rev	GGTCTCGCAGTTGCGCCC
pQE30 hNotch-1 EGF23-25 cloning	
EGF23 Fwd	TAGTAGAGATCTATAGAAGGACGATCAGCAGTCGACATCAACGAGTGCG
EGF25 Rev	TAGTAGCTCGAGCTCACAGTGGATCCCGCTG
pQE30 hNotch-1 EGF20-23 cloning	
EGF20EK_F	TAGTAGGGATCCGATGACGATGACAAATCAGCAATCAACAACAACGAGTGTG
EGF23_R	TAGTAGAAGCTTCTATTAGGTCTCGCAGTTGCGCCCACTG
