# Supplementary material for: Structural and functional studies of the EGF20-27 region reveal new features of the human Notch receptor important for optimal activation
Source: Structure. 2024 Dec 5;32(12):2325–2336.e5. doi: 10.1016/j.str.2024.10.012 (PMC12979247; doi:10.1016/j.str.2024.10.012)
Supplement: Document S2. Article plus supplemental information [file mmc2.pdf]

Structure

Article

Structural and functional studies of the EGF20-27 region reveal new features of the human Notch receptor important for optimal activation

Graphical abstract

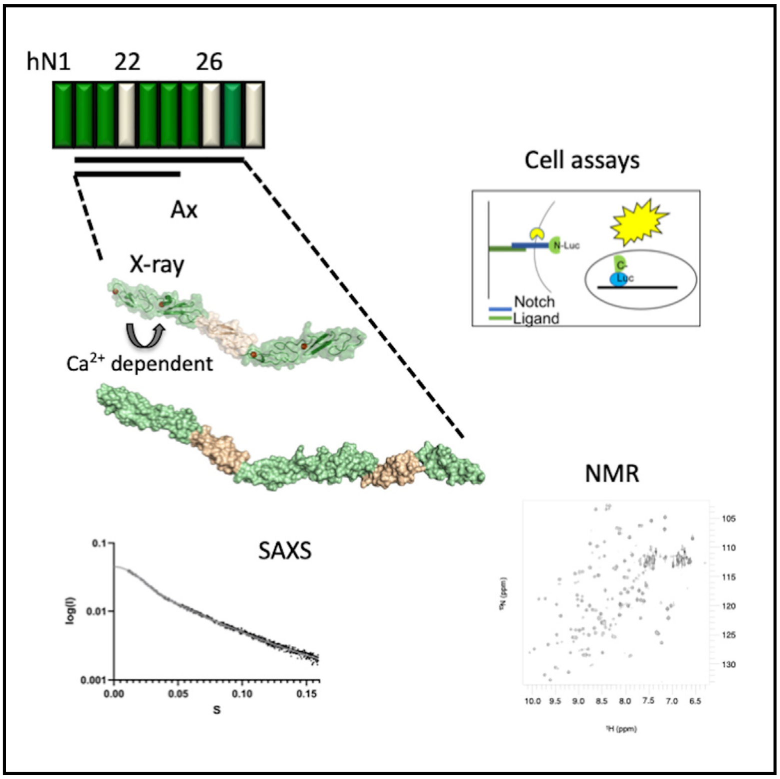

Authors

Zhihan Bo, Thomas Rowntree, Steven Johnson, ..., Susan M. Lea, Christina Redfield, Penny A. Handford

Correspondence

susan.lea@nih.gov (S.M.L.),
christina.redfield@bioch.ox.ac.uk (C.R.),
penny.handford@bioch.ox.ac.uk (P.A.H.)

In brief

The structure of the Notch ectodomain remains unsolved. Bo et al. identify an elongated Ca^{2+}-dependent structure for EGF 20–27 which includes the *Abruptex* (Ax) region. Loss of Ca^{2+}-dependent structure within this region leads to reduced *trans*-activation and *cis*-inhibition, indicating a role for rigidity in optimizing generation of the Notch signal.

Highlights

- hN-1 EGF20-27 is mainly rigid and elongated with high Ca^{2+} affinity in most domains
- EGF21 has a weaker affinity for Ca^{2+} so the EGF20-21 interface may vary in rigidity
- EGF24-25 Ca^{2+}-binding site mutagenesis reduces *trans*-activation and *cis*-inhibition
- This indicates the functional importance of the Ca^{2+}-stabilized structure

Bo et al., 2024, Structure *32*, 2325–2336
December 5, 2024 © 2024 The Author(s). Published by Elsevier Inc.
https://doi.org/10.1016/j.str.2024.10.012

CellPress

Structure

CellPress
OPEN ACCESS

Article

Structural and functional studies of the EGF20-27 region reveal new features of the human Notch receptor important for optimal activation

Zhihan Bo,[1,3] Thomas Rowntree,[1,3] Steven Johnson,[2,3,4] Hilman Nurmahdi,[1] Richard J. Suckling,[2] Johan Hill,[1] Boguslawa Korona,[1] Philip C. Weisshuhn,[1] Devon Sheppard,[2] Yao Meng,[1] Shaoyan Liang,[1] Edward D. Lowe,[1] Susan M. Lea,[2,4,*] Christina Redfield,[1,*] and Penny A. Handford[1,5,*]

[1]Department of Biochemistry, University of Oxford, South Parks Road, Oxford OX1 3QU, UK

[2]Sir William Dunn School of Pathology, University of Oxford, South Parks Road, Oxford OX1 3RE, UK

[3]These authors contributed equally

[4]Present address: Center for Cancer Research, National Cancer Institute, Frederick, MD 21702

[5]Lead contact

*Correspondence: susan.lea@nih.gov (S.M.L.), christina.redfield@bioch.ox.ac.uk (C.R.), penny.handford@bioch.ox.ac.uk (P.A.H.)

https://doi.org/10.1016/j.str.2024.10.012

SUMMARY

The Notch receptor is activated by the Delta/Serrate/Lag-2 (DSL) family of ligands. The organization of the extracellular signaling complex is unknown, although structures of Notch/ligand complexes comprising the ligand-binding region (LBR), and negative regulatory region (NRR) region, have been solved. Here, we investigate the human Notch-1 epidermal growth factor-like (EGF) 20-27 region, located between the LBR and NRR, and incorporating the Abruptex (Ax) region, associated with distinctive *Drosophila* phenotypes. Our analyses, using crystallography, NMR and small angle X-ray scattering (SAXS), support a rigid, elongated organization for EGF20-27 with the EGF20-21 linkage showing Ca^{2+}-dependent flexibility. In functional assays, Notch-1 variants containing Ax substitutions result in reduced ligand-dependent *trans*-activation. When *cis*-JAG1 was expressed, Notch activity differences between WT and Ca^{2+}-binding Ax variants were less marked than seen in the *trans*-activation assays alone, consistent with disruption of *cis*-inhibition. These data indicate the importance of Ca^{2+}-stabilized structure and suggest the balance of *cis*- and *trans*-interactions explains the effects of *Drosophila Ax* mutations.

INTRODUCTION

The Notch receptor is part of a core metazoan signal transduction pathway which performs crucial roles during development and in the adult organism including cell-fate determination, cell proliferation, and apoptosis and has an impact on most tissues and organs.[1,2] In adults, Notch has key roles in tissue homeostasis by regulating stem cell maintenance and function, immune system activation, and angiogenesis. Dysregulation of the Notch pathway results in both inherited and acquired disease, including many cancers, but also occurs during aging in normal esophageal epithelial tissue where hNotch-1 mutations drive clonal expansion and appear protective against tumorigenesis.[3,4]

Canonical Notch signaling requires cell-surface expression of a hetero-dimeric *trans*-membrane receptor which is extensively modified by O-glucosylation and O-fucosylation.[5–8] Ligand binding by one of the Jagged/Serrate or Delta (DLL) families, followed by ligand endocytosis, exposes the S2 site within the negative regulatory region (NRR) of Notch to proteolytic cleavage by ADAM10. Following this, γ secretase cleaves at the intramembrane S3 site releasing the intracellular domain of Notch (NICD).[9,10] Subsequently, NICD translocates to the nucleus, and in the presence of mastermind-like (MAML) binds to a transcription factor of the CBF1, suppressor of hairless, Lag-1 (CSL/RBPJk) family, and relieves repression of genes of the HES and Hey families.[11] Notch ligand activity is sensitive to the modification of O-fucosylated Notch by Fringe.[12–15] This can modulate signaling by different ligands important in controlling embryonic patterning and boundary formation between adjacent developmental compartments.

Although the downstream consequences of Notch receptor activation have been extensively studied, there is less knowledge about the initial cell-cell mediated extracellular Notch/ligand binding event and how this is converted into an activating signal. Identification of an N-terminal C2 domain in the canonical ligands suggested lipid binding at the cell surface may be important for optimal activation.[16–18] Different activation dynamics which result in opposing effects on myogenic cell fate were shown to be mediated by DLL1 and DLL4 signaling.[19] Data have also shown *cis*-activation to be an additional mode of Notch signaling together with *cis*-inhibition and *trans*-activation.[20] These and other studies underscore the importance of

© 2024 The Author(s). Published by Elsevier Inc.
This is an open access article under the CC BY license (http://creativecommons.org/licenses/by/4.0/).

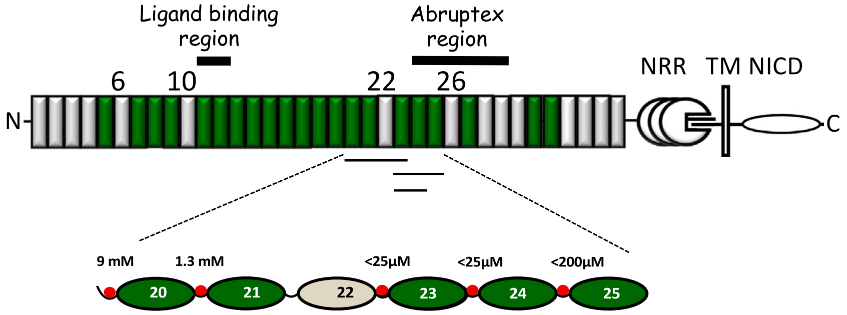

Figure 1. Modular organization of the extracellular domain of hNotch-1 and overview of Ca^{2+} dissociation constants

(Upper panel) The negative regulatory region (NRR) and transmembrane domain (TM) of hNotch-1 are indicated. Individual domains belonging to the Notch intracellular domain (NICD) are not indicated separately. Ca^{2+}-binding and non-Ca^{2+}-binding EGF domains are indicated in green and wheat, respectively. The thick horizontal black lines highlight the ligand-binding region, EGF11-13, and the Abruptex region, EGF24-29. The thin black lines indicate the constructs used for Ca^{2+} dissociation constant determination (EGF20-23, EGF23-25, EGF23-24).

(Lower panel) The measured Ca^{2+} dissociation constants at pH 7.5 and I = 0.15 for the EGF20-25 region are shown. K_D values were determined by NMR. Ca^{2+} is indicated by a red circle at the N terminus of each Ca^{2+}-binding EGF domain. The weak affinity for EGF20 reflects its non-native context in EGF20-23 where it lacks a preceding EGF domain which would be expected to stabilize the Ca^{2+}-bound state (also see Figures S1–S4).

understanding the pleiotropy of receptor/ligand structures and interactions at the cell surface and their subsequent fate.

High-resolution structures of receptor and ligand fragments, both in isolation and in complex, have been determined giving insight into the core interaction surfaces and the role of O-glycans.[16,18,21–26] Further *in vivo* and *in vitro* work has begun to decipher the complex Fringe code and how this regulates Notch EGF domain sensitivity to different ligands.[15,27] The structure of the NRR has been determined establishing its mechanosensory role in Notch signal activation which regulates S2 cleavage.[28,29] However, there has been much less structural information available for other regions of the extracellular portion of Notch, which are mainly comprised of multiple tandem repeats of EGF domains (Figure 1). We previously identified both flexible and rigid EGF-like domain interfaces in the N-terminal EGF4-13 extracellular region of hNotch-1 which binds ligand.[30] These latter data reveal that this portion of the receptor does not simply extend from the cell surface in a near-linear rigid fashion, as once predicted, but has the potential to adopt a range of conformations. This is supported by a mass spectrometry/small angle X-ray scattering (SAXS) study which showed that Notch and ligand ectodomains can form novel intra- and inter-molecular interactions, incompatible with a linear rod-like structure.[31]

In this study, we have used X-ray crystallography, NMR spectroscopy, and SAXS to investigate the structure and flexibility of the EGF20-27 region of the hNotch-1 ectodomain by analyzing a series of limited fragments (Figure 1). This region is homologous to *Drosophila* Notch where *Abruptex* (*Ax*) missense mutations in the EGF24-29 region result in specific phenotypes which are distinct from Notch null or loss of function phenotypes.[32–35] We report crystal structures of EGF21-23 and EGF20-24, where the domain interface formed between the non-Ca^{2+}-binding domain, EGF22, and its preceding domain introduces a near-linear conformation to the region. Ca^{2+}-binding measurements identify high-affinity sites (K_D<25 μM) for EGF23 and 24 domains in the 20–24 region and an unexpectedly low affinity site in EGF21 (K_D~1.3 mM). The dynamic behavior of the EGF20-21 interface has been probed as a function of Ca^{2+} concentration using {^{1}H}-^{15}N heteronuclear Nuclear Overhauser Effect (NOE) measurements and residual dipolar couplings; these data suggest that changes in local Ca^{2+} concentrations may modulate the flexibility of this region. Data from SAXS analysis of the EGF20-27 construct have been used, together with the EGF20-24 X-ray structure, to model the overall topology of this region which appears rigid and elongated. In parallel, functional studies of full-length Notch-1 variants revealed lower ligand-mediated Notch *trans*-activation in cellular assays either due to defective Ca^{2+} binding to EGF24 and EGF25 or misfolding. Observed differences in ligand-dependent activation of Ca^{2+} binding Notch variants and WT were suppressed when JAG1 was expressed in *cis*, within the same cell as receptor. These data are consistent with an important role for the Ca^{2+}-dependent structure of the EGF20-27 region in facilitating ligand-dependent activation and *cis*-inhibition and provide a plausible explanation for a subset of unusual *Abruptex* phenotypes.

RESULTS

Crystal structures of hNotch-1 EGF21-23 and EGF20-24 reveal a near-linear conformation

Structures of hNotch-1 EGF21-23 and hNotch-1 EGF20-24 were determined using X-ray crystallography to resolutions of 1.55 Å and 1.50 Å respectively (Table 1). The structures are elongated, with each domain within the construct displaying a canonical EGF fold (Figure 2). Tilt and twist angles between adjacent domains pairs in hNotch-1 EGF20-24 are listed in Table 2, and hNotch-1 EGF21-23 superposes onto hNotch-1 EGF20-24 with an r.m.s.d < 1 Å (over 111 C_α atoms).

As expected, EGF20, EGF21, EGF23, and EGF24 each have a single Ca^{2+} bound and show modification by O-fucose and O-glucose. Ca^{2+} occupancy of the EGF20 site was only 45%; this is likely due to the weak affinity of this non-native N-terminal site (see in the following text).[36–38] EGF24 is unusual since the consensus aromatic residue F930, located between the 3rd and 4th cysteine residues and usually involved in stabilizing intramolecular structure, is shifted by one residue (+1) and points out into solvent. It may therefore be available for intermolecular interactions. This feature is specific to mammalian Notch-1 (human, mouse, and rat) and is not seen in Notch-2 (human, mouse, and rat) or *Drosophila* Notch, all of which have the same conserved 36 EGF domain modular organization. EGF22 does not contain a Ca^{2+}-binding consensus sequence. The structure shows a rigid and extended relative orientation of the Ca^{2+}-binding EGF21 and non-Ca^{2+}-binding EGF22 with a tilt angle of only 13.4°. This is in contrast to other Notch Ca^{2+}-binding EGF-non-Ca^{2+}-binding EGF pairs (~90° bent structure observed

CellPress
OPEN ACCESS

Table 1. Crystallization and structure determination

	hNotch-1 EGF21-23	hNotch-1 EGF20-24
Data collection		
Beamline	Diamond I04-1	Diamond I03
Space group	$P4_1$	P1
Wavelength (Å)	0.92	0.9763
Cell dimensions (Å)		
a, b, c (Å)	41.9, 41.9, 61.2	28.0, 35.1, 63.9
α, β, γ (°)	90.0, 90.0, 90.0	99.7, 92.4, 96.9
Resolution range (Å)[a]	41.92–1.55 (1.59–1.55)	62.9–1.50 (1.53–1.50)
Unique reflections	15359 (1109)	36167 (2764)
R_{merge}[a,b]	0.022 (0.497)	0.056 (0.483)
R_{meas}[a,c]	0.040 (0.667)	0.068 (0.602)
$CC_{1/2}$[a,d]	0.999 (0.834)	0.996 (0.884)
Mean $I/\sigma I$[a]	27.1 (2.5)	7.7 (1.7)
Completeness (%)[a]	99.7 (99.5)	95.2 (94.4)
Multiplicity[a]	4.7 (4.7)	2.6 (2.6)
Anomalous completeness	93.8 (93.1)	–
Anomalous multiplicity	2.2 (2.1)	–
Anomalous correlation	0.721 (0.028)	–
Wilson <B> ($Å^2$)	27.7	20.7
Refinement		
Resolution range (Å)[a]	41.9–1.55 (1.67–1.55)	62.9–1.50 (1.54–1.50)
No. of reflections	15331	36167
R_{work}/R_{free}[a]	0.195/0.218 (0.316/0.366)	0.175/0.197 (0.294/0.319)
Number of atoms/B-factors ($Å^2$)		
Protein	841/45.6	1439/32.6
Ligand/ion	2/24.0	83/42.3
Water	93/50.4	288/42.5
Rmsd from ideal values		
Bond lengths (Å)	0.004	0.014
Bond angles (°)	0.670	1.46
Ramachandran plot		
Favored region (%)	99.1	97.9
Allowed (%)	100.0	100.0
Outliers (%)	0	0
Rotamer outliers (%)	1.0	0.6
C-beta outliers	0	0
PDB ID code	9B3G	9B3N

[a]Values in parentheses are for the highest resolution shell.
[b]$R_{merge} = \Sigma(I_{hl} - \langle I_h \rangle)/\Sigma(I_{hl})$ where $\langle I_h \rangle$ is the mean intensity of unique reflection *h*, summed over all reflections for each observed intensity I_{hl}.
[c]$R_{meas} = \Sigma(n/n-1)^{1/2}(I_{hl} - \langle I_h \rangle)/\Sigma(I_{hl})$ where *n* is the number of observations for unique reflection *h* with mean intensity $\langle I_h \rangle$, summed over all reflections for each observed intensity I_{hl}.
[d]$CC_{1/2}$ is the correlation coefficient on $\langle I \rangle$ between random halves of the dataset.

previously for EGF5-6, and the flexible interface observed in solution between EGF9-10[30]).

Ca^{2+}-binding measurements reveal rigid interfaces for Ca^{2+}-binding EGF domains in the EGF22-25 region of hNotch-1 but a flexible interface at EGF20-21

Ca^{2+} affinities for Ca^{2+}-binding (cb) EGF domains of the EGF20-25 region of human Notch-1 were measured to gain insight into the rigidity of interdomain interfaces in this region in solution. In EGF domains, a consensus sequence of D/N/E-x-D/N-D/N/E/Q-x_m-D/N/Q*-x_n-Y/F (where * indicates possible β-hydroxylation, and m/n are variable) is predictive for Ca^{2+} binding[36–40] and a high affinity for Ca^{2+}-binding is correlated with a well-defined and rigid domain interface.[30,41] Ca^{2+}-binding EGF domains 20, 21, 23, and 25 show the consensus Ca^{2+} binding sequence and the aromatic residue in the preceding domains

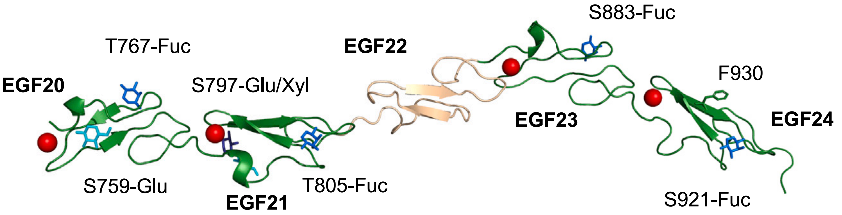

Figure 2. Structure of hNotch-1 EGF20–24 reveals an extended conformation

X-ray structure of EGF20–24 is shown in a cartoon representation. Ca^{2+}-binding EGF domains are shown in green, while the non-Ca^{2+}-binding EGF domain is shown in wheat. The Ca^{2+} ions bound in EGF20, EGF21, EGF23, and EGF24 are shown as red spheres. Ser/Thr residues glycosylated in the S2 expression system are labeled; glucose, fucose and xylose are shown in cyan, mid-blue and dark-blue respectively. The unusual solvent exposed position of F930 in EGF24 is shown in green.

which contributes to a hydrophobic packing interaction seen in many cbEGF/cbEGF and EGF/cbEGF rigid interfaces.

NMR titrations using ^{15}N-labeled EGF20-23 and EGF23-24 were used to measure K_D values for the EGF20, EGF21, EGF23, and EGF24 sites (Figure S1).[30,42] 1D ^{1}H NMR was used to estimate K_D values for EGF25 in the EGF23-25 construct. The measured Ca^{2+} affinities are summarized in Figure 1. The K_D values for EGF domains 23, 24, and 25 are in the range of ~25–~200 μM and under the conditions of extracellular free Ca^{2+} concentration (~1.5 mM) and physiological ionic strength (I = 0.15) at pH 7.4 these sites will be saturated to >~95%. EGF21 has a significantly weaker affinity for Ca^{2+} (K_D ~1.3 mM), despite its native context; this site will only be occupied in ~50% of molecules. The high Ca^{2+} affinity observed for all the cbEGF domains except EGF21 suggests that the cbEGF domains from 23 to 25 form a packing interaction in solution with the preceding domain leading to a rigid interdomain interface.

Two *Ax* missense mutations identified in EGF24 (D948V) and EGF25 (N986I) of *Drosophila* Notch were investigated for their effects on Ca^{2+} binding in EGF pair or triple domain constructs (Figures S2 and S3). Each substitution reduced the Ca^{2+} binding affinity of the mutant domain without impacting the affinity of the neighboring domain. To test the impact of homologous mutations in hNotch-1, D909V was introduced into EGF24 in EGF23-24 and EGF23-25 constructs and reduced the Ca^{2+} binding affinity of the mutant domain but not adjacent domains (Figure S4). Each of these mutations therefore appears to act by introducing flexibility at the 23–24 or 24–25 interfaces.

Table 2. Interdomain tilt and twist angles observed in the X-ray structure of hNotch-1 EGF20-24

Domain Pair	Tilt Angle[a]	Twist Angle[a]
20–21	5.5°	135.2°
21–22	13.4°	51.1°
22–23	43.1°	171.1°
23–24	32.1°	156.3°

[a]The program mod2[62,63] was used to measure the tilt and twist angles for each pair of EGF domains. The tilt angle informs on the linearity of the domain pair; a value close to 0° is expected for a linear and elongated domain pair while a value close to 90° is expected for a pair oriented at right angles to each other. The twist angle informs on the rotation along the interdomain axis of one domain relative to the other and can be most easily visualized by the relative rotation of the anti-parallel β sheets in the two domains.

Dynamics in solution

Heteronuclear NOE shows Ca^{2+}-dependent dynamics in EGF21

The {^{1}H}-^{15}N heteronuclear NOE provides a method for identifying regions of the polypeptide backbone that undergo fast timescale dynamics (ps/ns).[43] Data for the EGF20-23 construct at three different Ca^{2+} concentrations (1.4 mM, 2.8 mM, and 40 mM $CaCl_2$) are shown in Figure 3A; under these conditions the occupancy of each Ca^{2+} binding site will differ. Reduced values of the heteronuclear NOE, characteristic of mobile residues, were observed for the first few residues at the N terminus of EGF20 and for several residues between the 3rd and 4th cysteine of EGF20. These residues are involved in Ca^{2+} binding and their mobility reflects the weak affinity of this domain for Ca^{2+} in its non-native context. Interestingly, EGF21, which is placed in a native context, showed similar reduced NOE values for some residues in the region between the 3rd and 4th cysteines at 1.4 mM $CaCl_2$, when the domain does not have Ca^{2+} bound (~23% saturated). The flexibility of these residues decreased in 2.8 mM Ca^{2+} when the site is ~55% occupied. These residues became rigid in 40 mM Ca^{2+} when the domain is fully bound with Ca^{2+}. As expected EGF23 had a rigid backbone, consistent with full occupancy of its Ca^{2+} binding site at each concentration. EGF22, which lacks a Ca^{2+} binding site, also had a rigid backbone under all conditions studied. With a K_D of 1.3 mM, the backbone flexibility observed in EGF21 could be functionally relevant at physiological Ca^{2+} concentrations.

Residual dipolar couplings show Ca^{2+}-dependent interdomain orientations

Residual dipolar couplings (RDCs) are a useful NMR parameter for assessing the relative orientations of protein domains in solution and for identifying interdomain dynamics on a wider range of timescales than the heteronuclear NOE.[44–48] We have used RDCs previously to define interfaces in the EGF4-EGF13 region of hNotch-1.[30] $^1H^N$-^{15}N RDCs were measured for EGF20-23 at two Ca^{2+} concentrations (low-0.84 mM and high-40 mM $CaCl_2$) using 2% C12E6/*n*-hexanol as the alignment medium.[49] It can be seen in Figure 3B that the magnitude of the RDCs observed in all four EGF domains increases as the Ca^{2+} concentration changes from low to high. This indicates stronger alignment of EGF20-23 resulting from a more elongated and rigid structure at the higher Ca^{2+} concentration. Fits of the RDCs for EGF22 and EGF23 at both Ca^{2+} concentrations, using the method we described previously,[30] yield interdomain tilt and twist angles that are consistent with those observed in the X-ray structure indicating a rigid interface between EGF22 and EGF23 in solution. At the lower Ca^{2+} concentration the RDCs observed for

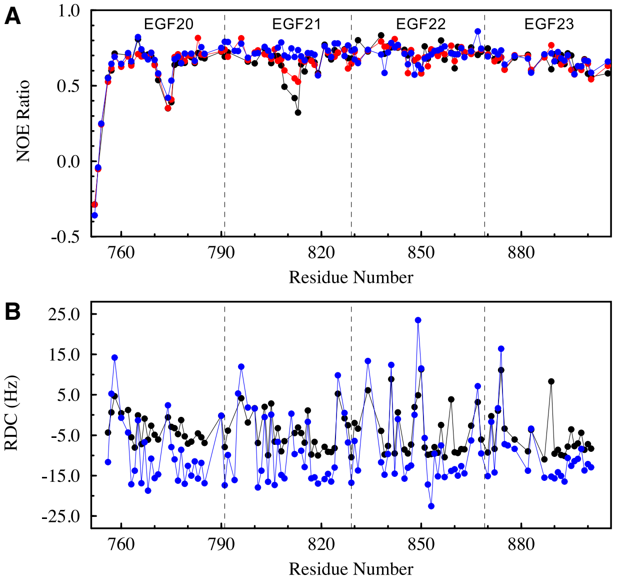

Figure 3. NMR measurements of Ca^{2+}-dependent dynamics in EGF20-23

(A) {^{1}H}-^{15}N heteronuclear NOE data for hNotch-1 EGF20-23 measured at Ca^{2+} concentrations of 1.4 mM (black circle), 2.8 mM (red circle) and 40 mM (blue circle) $CaCl_2$. Based on experimental K_D measurements (Figure 1), the EGF23 site will be fully occupied with Ca^{2+} at all three concentrations. The EGF21 site will be 23%, 55%, and 100% occupied, while the EGF20 site will be 4%, 14%, and 75% occupied. Reduced NOE ratios, characteristic of significant mobility on a nanosecond to picosecond timescale, are observed at the N terminus of EGF20 and for several residues between the 3rd and 4th cysteine of EGF20. EGF21 shows Ca^{2+}-dependent NOE values for some residues in the region between the 3rd and 4th cysteines. These residues become rigid in 40 mM Ca^{2+} when the domain is fully bound with Ca^{2+}. EGF23, which has Ca^{2+} bound in all three experiments has a rigid backbone. The dashed vertical lines represent the boundary between EGF domains.

(B) Residual dipolar couplings (RDCs) measured for 540 μM hNotch-1 EGF20-23 at low (0.84 mM) (black circle) and high (40 mM) (blue circle) Ca^{2+} concentrations. Based on experimental K_D measurements (Figure 1), EGF23 will be fully occupied at both concentrations, EGF21 will be 15% and 100% occupied and EGF20 will be 2% and 75% occupied. The magnitude of the RDCs observed in all EGF domains increases as the Ca^{2+} concentration increases from 0.84 mM to 40 mM. This is demonstrated by the large and similar values of 15.4, 16.9, 14.1, and 14.8 obtained for D_a, the axial component of the alignment tensor, for EGF20, EGF21, EGF22, and EGF23, respectively, at 40 mM $CaCl_2$ which indicate an elongated and rigid structure. At 0.84 mM $CaCl_2$, D_a values of 6.2, 9.0, 9.5, and 9.2 are obtained for EGF20, EGF21, EGF22, and EGF23, respectively. The lower values observed indicate a less elongated structure. The significantly lower value for EGF20 reflects the flexibility that exists between the EGF20 and EGF21 domains when the Ca^{2+}-binding site in EGF21 is not fully occupied.

EGF20 are of a smaller magnitude than those observed for EGF21, EGF22, and EGF23. This reflects the flexibility that exists between the EGF20 and EGF21 domains when the Ca^{2+}-binding site in EGF21 is not fully occupied. The RDC data are consistent with a Ca^{2+}-dependent interface between EGF20 and EGF21, as identified previously using the heteronuclear NOE.

SAXS analysis

We were unable to obtain diffraction-quality crystals of hNotch-1 EGF20-27. To determine the overall shape of this region and to assess possible flexibility in solution, SAXS measurements were collected on a sample resolved by SEC in 5mM Tris, pH 7.5, 15 mM Ca^{2+} (Table 3). The shape of the scattering curve (Figure 4A) shows that EGF20-27 is non-globular, as expected for this multi-domain construct, while the Kratky plot (Figure 4B) demonstrates that EGF20-27 has an extended rather than an unfolded structure. The P(r) distribution (Figure 4C) is also consistent with an extended structure for this 8-domain construct and shows a D_{max} of 238 Å for EGF20-27; a completely extended model for an 8-domain construct would be expected to have a D_{max} of ~240 Å based on a length of ~30 Å per EGF domain. A model consisting of the EGF20-24 X-ray structure and single EGF domain structures for EGFs 25, 26 and 27 was refined against SAXS data for EGF20-27 using SREFLEX from the ATSAS suite[50] and shows an extended and near linear conformation (Figure 4D). A similar SAXS analysis was carried out for a smaller EGF23-27 construct and is also consistent with an extended structure for EGF23-27 with a shorter D_{max} of 160 Å determined from the P(r) distribution (Figure S5). In addition, we have used AlphaFold2 (see STAR Methods) to generate a model for EGF20-27. This shows an extended structure that is similar to that generated by SREFLEX but the latter model gives better agreement with experimental SAXS data (Figure S6).

Table 3. SAXS data collection and processing parameters for hNotch-1 EGF20-27

Data collection parameters	
Instrument	Diamond Light Source
Beamline	B21
Wavelength (Å)	0.9537
q-range (Å $^{-1}$)	0.0032–0.38
Sample-to-detector distance (m)	3.6
Experiment	SEC-SAXS
Exposure time per frame (sec)	1
Temperature (°C)	20
Detector	Eiger 4M
Structural parameters	
I_0 (cm^{-1}) [from Guinier]	0.043
Rg (Å) [from Guinier]	49.61 ± 0.25
sRg limits (nm)	0.62–1.3
Dmax (Å) [Gnome]	238
I_0 (cm^{-1}) [from p(r)]	0.04
Rg (Å) [from p(r)]	56.10
Bayesian Molecular mass (kDa) [Primus]	53.1
Calculated Mw from sequence (kDa)	32.4

Ligand-dependent activation of Notch is reduced by substitutions in the EGF24-26 region

The functional importance of the EGF20-27 region was investigated by comparing the JAG1 and DLL4-dependent activity of full-length Notch-1 variants using a well-established luciferase complementation assay.[51] These included variants with single Ca^{2+} binding consensus substitutions D909V in EGF24, N947I in EGF25 (homologous to *Drosophila Ax* suppressor variants) and a DVNI double variant, C933S (EGF24) and C960Y (EGF25) (homologous to *Drosophila Ax* homozygous lethal

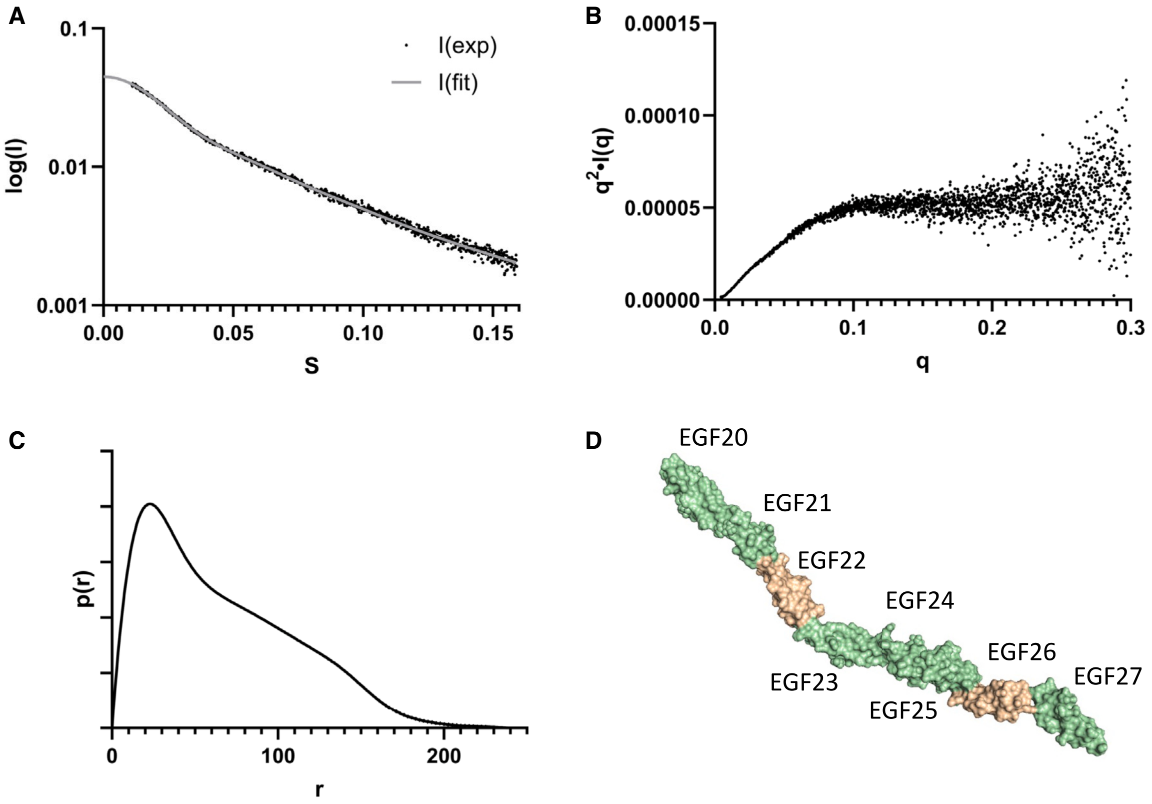

Figure 4. SAXS data for hNotch-1 EGF20-27 reveal an extended shape

(A) A scaled, merged, and averaged X-ray scattering curve was collected with purified hNotch-1 EGF20-27 at 2.5 mg/mL in 5mM Tris, pH 7.5, 15 mM $CaCl_2$.

(B and C) (B) Kratky plot and (C) P(r) distribution derived from the scatter curves collected from purified hNotch-1 EGF20-27.

(D) Model for EGF20-27 derived from the SAXS data using SREFLEX refinement (also see Figures S5 and S6). Ca^{2+}-binding and non-Ca^{2+}-binding domains are indicated in green and wheat respectively.

variants) and S990N (EGF26) a substitution commonly found in aging esophageal tissue (Figure 5A).[4] An EGF12 L468A substitution, which has previously been shown to be critical for ligand binding,[52] was also tested and acted as a negative control. Initially both an EGTA-induced ligand-independent activation assay, and direct measurement of cell surface Notch levels were used to demonstrate similar expression levels of mutant receptor on the cell surface compared to WT for D909V, N947I, and DVNI, and the control L468A variant (Figure S7). However C933S, C960Y, and S990N all showed reduced cell surface levels. In ligand-dependent assays, either utilizing cells expressing full-length mouse ligands (Jagged1 or Dll-4) or plate-bound purified human ligand fragments (JAG1 or DLL4), variants D909V, N947I, and DVNI all reduced the ability of Notch-1 to be *trans*-activated in response to both ligands, (Figure 5B; Figure S7). This reduction in activation was not as pronounced as that observed for L468A (Figure 5B) but nonetheless indicated an important role for the Ca^{2+} dependent structure of this region. As expected C933S and C960Y both showed reduced activity consistent with lower expression levels on the cell surface, most likely as a consequence of misfolding due to loss of a key disulphide bond. Thus the homozygous lethality associated with a subset of *Drosophila Ax* mutations, may be explained by a substantial quantitative defect of Notch at the cell surface. Variant S990N also showed reduced activity consistent with low cell surface levels. This may be due to defective O-glucosylation since modification of Ser/Thr residues between C1 and C2 within Notch EGF domains is known to facilitate folding (although S990, located at +3 from C1 within this loop region does not conform to the defined consensus).[53] However the asparagine substitution at residue 990 creates an NxC motif which can be associated with N-glycosylation.[54] Thus it is also possible that incorrect addition of an N-glycan to EGF26 impedes folding. The quantitative defect associated with this substitution further supports the hypothesis that loss of function Notch mutations accumulate in aging esophageal tissue.[4]

Cis-inhibition of Notch-1 requires native Ca^{2+}-dependent structure of EGF24-25

D909V, N947I, and DVNI variants were also tested in a *cis*-inhibition assay where each Notch variant cell line was transiently transfected with a construct expressing full-length JAG1 on the same membrane and challenged with cell surface-expressed ligands on B16 cells (Figures 5A, 5C, and S7). Lower levels of Notch activation were seen when WT JAG1 was expressed in the same cell as WT Notch-1, indicative of *cis*-inhibition. This was a direct effect of ligand ectodomain on Notch ligand-binding region (LBR) since the lower receptor activity observed was reversed by introduction of an F207A/E228K DSL JAG1 variant which disrupts the ligand/receptor interface (Figures 5C and S7).[17,25] The Notch variants,

CellPress
OPEN ACCESS

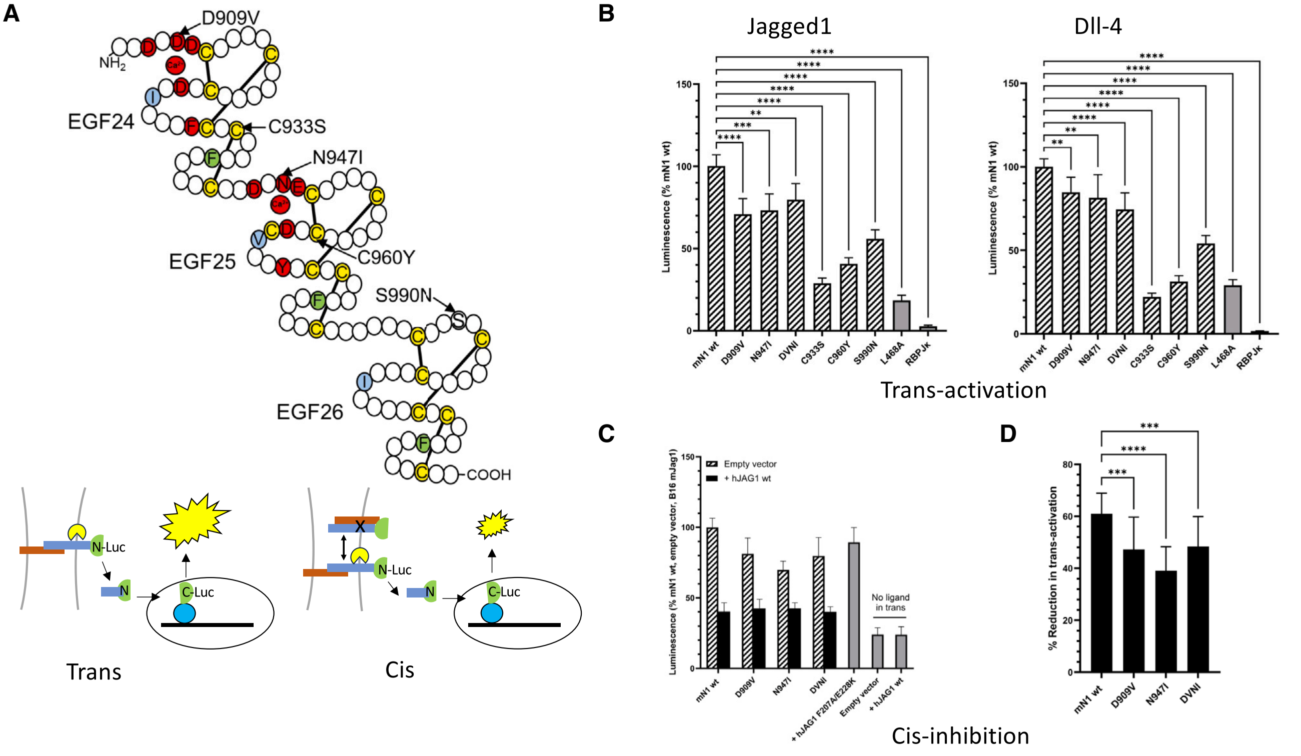

Figure 5. Luciferase reporter assays demonstrate Notch-1 variants from EGF24-26 region show defects in *trans*-activation and *cis*-inhibition

(A) (Upper panel) Amino acid substitutions from the Notch-1 EGF24-26 region studied. Conserved Cys residues indicated in yellow, Ca^{2+}-binding consensus residues shown in red, residues which form interdomain packing interactions shown in blue and green. (Lower panel) Cartoon of *trans*-activation and *cis*-inhibition cellular assays using the Notch luciferase reporter is shown. Ligand in red, Notch in blue. *Cis*-inhibition reduces the observed luminescence.

(B) Ligand-dependent *trans*-activation assays were performed with Notch-1 variant lines co-cultured with B16 cells expressing mJag1 (Jagged1) or mDll4 (Dll-4). Graphs show the combined results of four independent plates, each with five repeat wells. Signal in each case is shown relative to the WT Notch-1 (mN1 wt) line co-cultured with ligand. Data presented as mean ± SD. Statistical significance was determined by Kruskal-Wallis test and Dunn's post hoc test (** = $p < 0.01$, *** = $p < 0.001$, **** = $p < 0.0001$).

(C) *Cis*-inhibition of Notch-1 variant cells transfected with pcDNA3.1/full-length WT hJAG1. Controls were empty pcDNA3.1 or pcDNA3.1/hJAG1F207A/E228K (Notch-binding defective). Signal shown relative to WT Notch-1 (mN1 wt) transfected with empty pcDNA3.1 and co-cultured with B16 cells expressing mJag1. *Cis*-inhibition (black) is monitored indirectly as a decrease in luminescence signal arising from cells transfected with *cis*-JAG1 and shown alongside the luminescent signal obtained from cells transfected with empty vector (hatched). Data presented as mean ± SD.

(D) Percentage reduction in Notch *trans*-activation observed for *cis*-inhibited Ax variants (1-(% luminescence with *cis*-hJAG1)/(% luminescence empty vector)) ×100 based on data in (C). Data presented as mean ± SD. Statistical significance was determined by ordinary one-way ANOVA and Dunnett's post hoc test (*** = $p < 0.001$, **** = $p < 0.0001$) (also see Figures S7 and S8).

when similarly tested, also showed evidence of *cis*-inhibition, since the activity level of each cell line was reduced compared to that expressing empty plasmid alone. However, overall activity of variant and WT lines was very similar, once *cis*-inhibited (Figure 5C). Similar data were obtained irrespective of the ligand used to *trans*-activate the *cis*-inhibited cells (Figure S7). These data suggest there is also a negative effect of the Ca^{2+}-binding substitutions on the ability of Notch to be *cis*-inhibited (Figure 5D); otherwise the overall activity levels seen would be lower (reflecting the defect in *trans*-activation associated with these variants) (Figure 5B).

DISCUSSION

Although the Notch receptor was identified more than 30 years ago, the architecture of the large extracellular domain, which is crucial for understanding how Notch can form both activatory and inhibitory complexes with its ligands Delta and Serrate/Jagged, is still unknown. Up to now, high-resolution structural methods have failed to provide models for the full extracellular domain due to the difficulties of studying such a large extended and flexible molecule. Furthermore, electron microscopy (EM) studies have been largely unsuccessful at identifying receptor architecture due to problems associated with obtaining pure, natively folded protein (108 disulphide bonds in the EGF-domain-rich sections of Notch-1 alone) and the very narrow dimensions (in two directions) of this fibrous protein. Despite these difficulties, progress has been made by applying structural and biophysical methods to analyze multidomain fragments from different regions and combining these with functional studies. As a result, a structural model for hNotch-1 EGF4-13 has been produced which demonstrates that this region is not simply a rigid straight rod but has flexibility and nonlinear sections.[30] Using similar methods, combined with SAXS analysis, we focused on a region C-terminal to the LBR which in *Drosophila* gives rise to distinctive *Abruptex* phenotypes

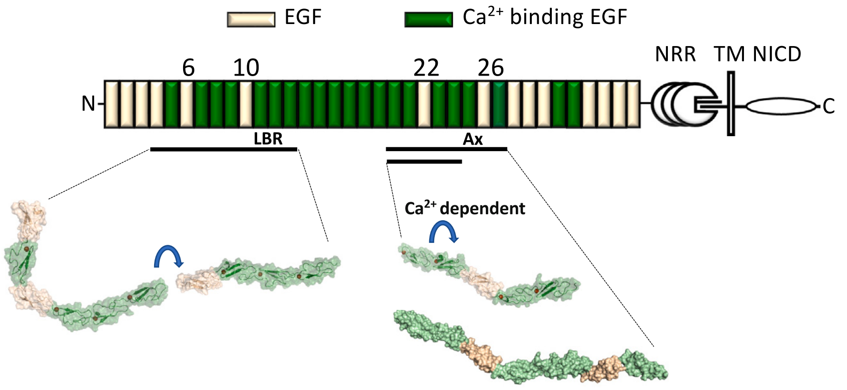

Figure 6. Structural model of the ectodomain of hNotch-1 comprising EGF domains

The previously published model of the EGF4-13 region is shown alongside that of the EGF20-27 region derived from the X-ray structure of EGF20-24 and SAXS data for EGF20-27. The flexible region determined by $\{^1H\}$-^{15}N heteronuclear NOE and RDC data is indicated with a blue arrow. The thick horizontal lines show the EGF4-13 region (containing the ligand-binding region (LBR) and the flexible interface between EGF9 and EGF10), the EGF20-24 region (determined by X-ray crystallography), and the EGF20-27 region (containing the Abruptex (Ax) region studied here and modeled by SAXS). Ca^{2+} and non-Ca^{2+}-EGF domains are indicated in green and wheat respectively.

when mutated, suggesting an important functional role in regulating Notch activity.[32]

Using X-ray crystallography, NMR, and SAXS analyses we describe a Ca^{2+}-dependent elongated structure for hNotch-1 EGF20-27 which is rigid in solution at high Ca^{2+} concentrations. Under conditions which mimic extracellular free [Ca^{2+}], we observe one weak affinity Ca^{2+} binding site in EGF21 which could confer flexibility at the EGF20/EGF21 interface. A structural model based on these data, together with that previously shown for EGF4-13, is shown in Figure 6. Full-length Notch variants with Ca^{2+}-binding substitutions in EGF24 and EGF25 show a reduced ability to be *trans*-activated and *cis*-inhibited in cell-based assays, demonstrating the importance of rigidity of EGF23/24 and EGF24/25 domain interfaces for these functions. Notch activity, when ligand is present in *cis* or *trans*, is dependent on the Notch LBR interacting with ligand DSL, as evidenced by the effects of ligand DSL substitutions F207A/E228K (Figures 5C, S7, and S8) which abrogate the known receptor/ligand interface. These data suggest that the DSL-EGF11 antiparallel interaction identified in high resolution structures of receptor/ligand complexes is common to both *cis*-inhibitory and *trans*-activatory complexes.

Significance of Ca^{2+} binding

A variety of methods has been used previously to identify the Ca^{2+}-binding affinity of a distinctive subset of EGF domains ranging from mM to nM dependent upon the domain context.[41] This measurement is of biological significance since the extracellular environment of EGF-rich proteins is high in Ca^{2+} and saturation of such sites, in EGF-cbEGF or cbEGF-cbEGF pairs usually confers rigidity to interfaces. This can facilitate Ca^{2+}-dependent protein-protein interactions (particularly where the interaction surface extends over a number of different adjacent domains), protect against proteolysis or confer biomechanical properties/resistance to pulling of the molecule in tissues subjected to tension.

In hNotch-1 at least 20 of the 36 domains are of the Ca^{2+}-binding type with a clearly defined set of residues known either to ligate Ca^{2+} directly or contribute to the stability of the Ca^{2+}-binding site. So far most sites studied in Notch-1 have exhibited affinity such that they would be saturated under physiological conditions (free Ca^{2+} ~1.5 mM $I = 0.15$, pH 7.4). However, unusually a weak affinity site was observed in EGF21 which imparted dynamic behavior on the EGF20-21 interface. The other sites determined here at 23, 24, and 25 (see Figure 1) would all be expected to be saturated under physiological conditions. In fibrillin-1, another extensively studied cbEGF-rich protein, only one similar weak site in the mM range has been identified (cbEGF32). Its functional significance is underscored by the discovery of a Marfan syndrome mutation resulting in a cbEGF32 N to S substitution, which by altering the geometry of Ca^{2+} ligation weakens the affinity 9-fold from ~1.6 mM to 14 mM.[55] Thus, the Ca^{2+}-binding affinity of EGF domains within a protein is fine-tuned for biological function. The flexibility at the hNotch-1 EGF20-21 domain interface conferred by a weak site may facilitate trafficking, protein-protein interactions in different contexts, contribute to catch bond behavior, or impart biomechanical properties. One other site of flexibility has previously been observed in hNotch-1 between EGF9 and 10.[30] In this case, EGF10 is non-Ca^{2+} binding and therefore the properties of this interface are Ca^{2+} independent. These sites of flexibility may explain the relatively short measurement for the Notch ectodomain calculated by SAXS in a previous study.[31]

Cis-trans interactions

It has been known for many years that *cis* and *trans* complexes of Notch with its ligands can form resulting in inhibitory/activatory effects on signaling. The mechanism by which such complexes could form when receptor and ligand were both membrane-bound, had asymmetrical binding sites and were apparently composed of rigid domain interfaces was difficult to envisage. Identification in this study of a Ca^{2+}-dependent pairwise linkage within contiguous EGF repeats, membrane proximal to the LBR, together with earlier identification of a flexible EGF9-10 interface, indicates that a variety of conformations are possible at the cell surface which might facilitate *cis* and *trans* interactions with ligand. For example, rotation about the EGF20-21 interface under physiological conditions could allow Notch to dock with ligand presented in *cis* (on the same cell surface) or in *trans* (on the opposing cell surface) while maintaining the same core ligand-binding interface. Flexible regions in receptors involved in *cis* and *trans* interactions with ligands which maintain the same binding site, such as Ly49 and MHC-I[56] and LILRB and MHC-I,[57] have previously been proposed. However other distinct modes of binding, such as seen in plexin receptors with semaphorins, can also occur.[58] The ability of hNotch-1 to sample a range of conformations may also allow it to dimerize on the same cell surface,

CellPress
OPEN ACCESS

form intramolecular interactions within the same monomer, and interact with ligands in *cis* and in *trans*.

Functional experiments

Functional data indicate that the Ca^{2+}-dependent conformation of the EGF24/EGF25 region which would be extended and rigid under physiological conditions is important for Notch-1 activation. Impairment of Ca^{2+} binding to these two domains does not impact on trafficking however as variants show similar levels of ligand-independent activation and cell surface expression (Figure S7). The location of this region between the LBR ligand interaction site and the NRR containing the S2 site cleaved by ADAM10 protease may help facilitate ligand binding in *cis* and *trans* but may also aid transmission of the pulling force required for S2 cleavage of the NRR mechanosensor. The Ax region has been observed previously to interact with the LBR of Notch suggesting that it may compete with ligand for this region.[59,60]

Dominant *Ax* missense mutations which affect the EG24-29 region in *Drosophila* Notch result in a shared phenotype, but can be distinguished by their behavior when crossed with Notch null alleles. The "suppressor" class of missense mutations affect EGF24 and EGF25 (and cause defective Ca^{2+} binding, Figure S3) while the "enhancer" class affect residues in C-terminal portions of EGF27 and 29, both of which are non-Ca^{2+}-binding domains. Our study indicates that loss of Ca^{2+}-stabilized interfaces at EGF23-24 and EGF24-25 underlies the suppressor phenotype and reduces the ability of Notch to be *trans*-activated and *cis*-inhibited. These cellular data suggest that the phenotypic consequences of such mutations may ultimately depend on the balance of *cis*- and *trans*- interactions that occur in a tissue context. When *trans* signals dominate, Notch activation may be reduced in the suppressor variants compared to WT. Where *cis*-inhibition occurs, activation may be similar to WT receptor. This may explain the observed loss of repression of Notch activity of Ax variants at the dorsal/ventral border in the *Drosophila* wing disc, compared to WT, when ligands Delta or Serrate are expressed ectopically.[61]

Overall, this study has increased our understanding of the architecture of the Notch ectodomain by providing information on the conformation of the EGF20-27 region. These data, coupled with earlier high- and low-resolution studies, show that the extracellular portion does have extended sections, but has at least two points of flexibility within it which allow the receptor to sample a range of conformations. Cellular experiments have demonstrated the importance of the Ca^{2+}-dependent rod-like structure of the EGF24-25 region for optimal *trans*-activation and *cis*-inhibition and thus establishes a mechanism for the effects of unusual phenotypes associated with *Abruptex* mutations.

RESOURCE AVAILABILITY

Lead contact

Requests for further information and further resources should be directed to, and will be fulfilled by the lead contact, Penny A Handford (penny.handford@bioch.ox.ac.uk).

Materials availability

Reagents generated in this study will be made available on request, but we may require a payment and/or a completed materials transfer agreement if there is potential for commercial application.

Data and code availability

- The coordinates of EGF21-23 and EGF20-24 have been deposited in the Protein Data Bank under accession numbers PDB: 9B3G and PDB: 9B3N.
- Resonance assignments for EGF20-23 have been deposited in the BioMagResBank (BMRB) under accession number BMRB: 51699. They are publicly available as of the date of publication. Accession numbers are also listed in the key resources table.
- All data reported in this paper will be shared by the lead contact on request.
- This paper does not report original code.
- Any additional information required to reanalyze the data reported in this paper is available from the lead contact upon request.

ACKNOWLEDGMENTS

This work was supported by MRC grant MR/R009317/1 and MR/V008935/1 awarded to P.A.H. S.M.L. was supported by a Wellcome Trust Investigator Award. J.H., T.R., and P.W. were supported by Wellcome Trust, BBSRC and Cancer Research UK studentships, respectively. We thank Abi Boyce and Lucy Barber for technical help. We thank the Diamond Light Source for access to SAXS and X-ray crystallography beamlines. HEK-RBP cells were a kind gift from R.Kopan, University of Cincinnati College of Medicine.

AUTHOR CONTRIBUTIONS

Protein constructs were expressed and purified by H.N., Z.B., P.C.W., S.L., R.J.S., J.H., and T.R.; T.R., J.H., P.C.W., S.L., and C.R. collected and analyzed Ca^{2+}-binding data. J.H. and C.R. collected and analyzed heteronuclear NOE and RDC data. T.R., H.N., Z.B., and E.D.L. collected and analyzed SAXS data. R.J.S., D.S., S.J. and S.M.L. collected and analyzed crystallographic data. Z.B., T.R., B.K., Y.M., and P.A.H. collected and analyzed Notch activation data. P.A.H., C.R., and S.M.L. conceived and supervised the research and wrote the manuscript. All authors discussed the results and implications of the data.

DECLARATION OF INTERESTS

The authors declare no competing interests.

STAR★METHODS

Detailed methods are provided in the online version of this paper and include the following:

- KEY RESOURCES TABLE
- EXPERIMENTAL MODEL AND STUDY PARTICIPANT DETAILS
  - Strains used in protein production
- METHOD DETAILS
  - Protein expression, purification, refolding and characterization
  - X-Ray crystallography
  - NMR spectroscopy
  - SAXS
  - HEK-RBP cells growth and transfection
  - B16 cells growth conditions
  - Notch activation assays
  - Flow cytometry-based cell surface Notch-1 quantification
- QUANTIFICATION AND STATISTICAL ANALYSIS

SUPPLEMENTAL INFORMATION

Supplemental information can be found online at https://doi.org/10.1016/j.str.2024.10.012.

Received: March 28, 2024
Revised: August 1, 2024
Accepted: October 8, 2024
Published: November 1, 2024

REFERENCES

1. Artavanis-Tsakonas, S., Rand, M.D., and Lake, R.J. (1999). Notch Signaling: Cell Fate Control and Signal Integration in Development. Science *284*, 770–776. https://doi.org/10.1126/science.284.5415.770.
2. Bray, S.J. (2006). Notch signalling: a simple pathway becomes complex. Nat. Rev. Mol. Cell Biol. *7*, 678–689. https://doi.org/10.1038/nrm2009.
3. Abby, E., Dentro, S.C., Hall, M.W.J., Fowler, J.C., Ong, S.H., Sood, R., Herms, A., Piedrafita, G., Abnizova, I., Siebel, C.W., et al. (2023). Notch1 mutations drive clonal expansion in normal esophageal epithelium but impair tumor growth. Nat. Genet. *55*, 232–245. https://doi.org/10.1038/s41588-022-01280-z.
4. Martincorena, I., Fowler, J.C., Wabik, A., Lawson, A.R.J., Abascal, F., Hall, M.W.J., Cagan, A., Murai, K., Mahbubani, K., Stratton, M.R., et al. (2018). Somatic mutant clones colonize the human esophagus with age. Science (New York, N.Y.) *362*, 911–917. https://doi.org/10.1126/science.aau3879.
5. Blaumueller, C.M., Qi, H., Zagouras, P., and Artavanis-Tsakonas, S. (1997). Intracellular cleavage of Notch leads to a heterodimeric receptor on the plasma membrane. Cell *90*, 281–291. https://doi.org/10.1016/S0092-8674(00)80336-0.
6. Logeat, F., Bessia, C., Brou, C., LeBail, O., Jarriault, S., Seidah, N.G., and Israël, A. (1998). The Notch1 receptor is cleaved constitutively by a furin-like convertase. Proc. Natl. Acad. Sci. USA *95*, 8108–8112. https://doi.org/10.1073/PNAS.95.14.8108.
7. Pandey, A., Niknejad, N., and Jafar-Nejad, H. (2021). Multifaceted regulation of Notch signaling by glycosylation. Glycobiology *31*, 8–28. https://doi.org/10.1093/glycob/cwaa049.
8. Sanchez-Irizarry, C., Carpenter, A.C., Weng, A.P., Pear, W.S., Aster, J.C., and Blacklow, S.C. (2004). Notch Subunit Heterodimerization and Prevention of Ligand-Independent Proteolytic Activation Depend, Respectively, on a Novel Domain and the LNR Repeats. Mol. Cell Biol. *24*, 9265–9273. https://doi.org/10.1128/mcb.24.21.9265-9273.2004.
9. Mumm, J.S., Schroeter, E.H., Saxena, M.T., Griesemer, A., Tian, X., Pan, D.J., Ray, W.J., and Kopan, R. (2000). A ligand-induced extracellular cleavage regulates gamma-secretase-like proteolytic activation of Notch1. Mol. Cell *5*, 197–206. https://doi.org/10.1016/s1097-2765(00)80416-5.
10. Schroeter, E.H., Kisslinger, J.A., and Kopan, R. (1998). Notch-1 signalling requires ligand-induced proteolytic release of intracellular domain. Nature *393*, 382–386. https://doi.org/10.1038/30756.
11. Jarriault, S., Brou, C., Logeat, F., Schroeter, E.H., Kopan, R., and Israel, A. (1995). Signalling downstream of activated mammalian Notch. Nature *377*, 355–358. https://doi.org/10.1038/377355a0.
12. Brückner, K., Perez, L., Clausen, H., and Cohen, S. (2000). Glycosyltransferase activity of Fringe modulates Notch–Delta interactions. Nature *406*, 411–415. https://doi.org/10.1038/35019075.
13. Haines, N., and Irvine, K.D. (2003). Glycosylation regulates Notch signalling. Nat. Rev. Mol. Cell Biol. *4*, 786–797. https://doi.org/10.1038/nrm1228.
14. Moloney, D.J., Panin, V.M., Johnston, S.H., Chen, J., Shao, L., Wilson, R., Wang, Y., Stanley, P., Irvine, K.D., Haltiwanger, R.S., and Vogt, T.F. (2000). Fringe is a glycosyltransferase that modifies Notch. Nature *406*, 369–375. https://doi.org/10.1038/35019000.
15. Pandey, A., Harvey, B.M., Lopez, M.F., Ito, A., Haltiwanger, R.S., and Jafar-Nejad, H. (2019). Glycosylation of Specific Notch EGF Repeats by O-Fut1 and Fringe Regulates Notch Signaling in Drosophila. Cell Rep. *29*, 2054–2066.e6. https://doi.org/10.1016/j.celrep.2019.10.027.
16. Chillakuri, C.R., Sheppard, D., Ilagan, M.X.G., Holt, L.R., Abbott, F., Liang, S., Kopan, R., Handford, P.A., and Lea, S.M. (2013). Structural Analysis Uncovers Lipid-Binding Properties of Notch Ligands. Cell Rep. *5*, 861–867. https://doi.org/10.1016/j.celrep.2013.10.029.
17. Meng, Y., Sanlidag, S., Jensen, S.A., Burnap, S.A., Struwe, W.B., Larsen, A.H., Feng, X., Mittal, S., Sansom, M.S.P., Sahlgren, C., and Handford, P.A. (2022). An N-glycan on the C2 domain of JAGGED1 is important for Notch activation. Sci. Signal. *15*, eabo3507. https://doi.org/10.1126/scisignal.abo3507.
18. Suckling, R.J., Korona, B., Whiteman, P., Chillakuri, C., Holt, L., Handford, P.A., and Lea, S.M. (2017). Structural and functional dissection of the interplay between lipid and Notch binding by human Notch ligands. EMBO J. *36*, 2204–2215. https://doi.org/10.15252/embj.201796632.
19. Nandagopal, N., Santat, L.A., LeBon, L., Sprinzak, D., Bronner, M.E., and Elowitz, M.B. (2018). Dynamic Ligand Discrimination in the Notch Signaling Pathway. Cell *172*, 869–880.e19. https://doi.org/10.1016/j.cell.2018.01.002.
20. Nandagopal, N., Santat, L.A., and Elowitz, M.B. (2019). Cis-activation in the Notch signaling pathway. Elife *8*, e37880. https://doi.org/10.7554/eLife.37880.
21. Cordle, J., Redfieldz, C., Stacey, M., van der Merwe, P.A., Willis, A.C., Champion, B.R., Hambleton, S., and Handford, P.A. (2008). Localization of the delta-like-1-binding site in human Notch-1 and its modulation by calcium affinity. J. Biol. Chem. *283*, 11785–11793. https://doi.org/10.1074/jbc.M708424200.
22. Cordle, J., Johnson, S., Tay, J.Z.Y., Roversi, P., Wilkin, M.B., de Madrid, B.H., Shimizu, H., Jensen, S., Whiteman, P., Jin, B., et al. (2008). A Conserved Face of the Jagged/Serrate DSL Domain is Involved in Notch Trans-Activation and Cis-Inhibition. Nat. Struct. Mol. Biol. *15*, 849–857. https://doi.org/10.1038/nsmb.1457.
23. Hambleton, S., Valeyev, N.V., Muranyi, A., Knott, V., Werner, J.M., McMichael, A.J., Handford, P.A., and Downing, A.K. (2004). Structural and functional properties of the human Notch-1 ligand binding region. Structure *12*, 2173–2183. https://doi.org/10.1016/j.str.2004.09.012.
24. Kershaw, N.J., Church, N.L., Griffin, M.D.W., Luo, C.S., Adams, T.E., and Burgess, A.W. (2015). Notch ligand delta-like1: X-ray crystal structure and binding affinity. Biochem. J. *468*, 159–166. https://doi.org/10.1042/BJ20150010.
25. Luca, V.C., Kim, B.C., Ge, C., Kakuda, S., Wu, D., Roein-Peikar, M., Haltiwanger, R.S., Zhu, C., Ha, T., and Garcia, K.C. (2017). Notch-Jagged complex structure implicates a catch bond in tuning ligand sensitivity. Science *355*, 1320–1324. https://doi.org/10.1126/science.aaf9739.
26. Luca, V.C., Jude, K.M., Pierce, N.W., Nachury, M.V., Fischer, S., and Garcia, K.C. (2015). Structural basis for Notch1 engagement of Delta-like 4. Science *347*, 847–853. https://doi.org/10.1126/science.1261093.
27. Kakuda, S., LoPilato, R.K., Ito, A., and Haltiwanger, R.S. (2020). Canonical Notch ligands and Fringes have distinct effects on NOTCH1 and NOTCH2. J. Biol. Chem. *295*, 14710–14722. https://doi.org/10.1074/jbc.RA120.014407.
28. Gordon, W.R., Roy, M., Vardar-Ulu, D., Garfinkel, M., Mansour, M.R., Aster, J.C., and Blacklow, S.C. (2009). Structure of the Notch1-negative regulatory region: implications for normal activation and pathogenic signaling in T-ALL. Blood *113*, 4381–4390. https://doi.org/10.1182/BLOOD-2008-08-174748.
29. Gordon, W.R., Vardar-Ulu, D., Histen, G., Sanchez-Irizarry, C., Aster, J.C., and Blacklow, S.C. (2007). Structural basis for autoinhibition of Notch. Nat. Struct. Mol. Biol. *14*, 295–300. https://doi.org/10.1038/nsmb1227.
30. Weisshuhn, P.C., Sheppard, D., Taylor, P., Whiteman, P., Lea, S.M., Handford, P.A., and Redfield, C. (2016). Non-Linear and Flexible Regions of the Human Notch1 Extracellular Domain Revealed by High-Resolution Structural Studies. Structure *24*, 555–566. https://doi.org/10.1016/j.str.2016.02.010.
31. Zeronian, M.R., Klykov, O., Portell i de Montserrat, J., Konijnenberg, M.J., Gaur, A., Scheltema, R.A., and Janssen, B.J.C. (2021). Notch–Jagged signaling complex defined by an interaction mosaic. Proc. Natl. Acad. Sci. USA *118*, e2102502118. https://doi.org/10.1073/pnas.2102502118.
32. de Celis, J.F., and Bray, S. (1997). Feed-back mechanisms affecting Notch activation at the dorsoventral boundary in the Drosophila wing. Development *124*, 3241–3251. https://doi.org/10.1242/dev.124.17.3241.

33. De Celis, J.F., and Garcia-Bellido, A. (1994). Modifications of the Notch function by Abruptex mutations in Drosophila melanogaster. Genetics *136*, 183–194. https://doi.org/10.1093/genetics/136.1.183.

34. Foster, G.G. (1975). Negative complementation at the notch locus of Drosophila melanogaster. Genetics *81*, 99–120. https://doi.org/10.1093/GENETICS/81.1.99.

35. Kelley, M.R., Kidd, S., Deutsch, W.A., and Young, M.W. (1987). Mutations altering the structure of epidermal growth factor-like coding sequences at the Drosophila Notch locus. Cell *51*, 539–548. https://doi.org/10.1016/0092-8674(87)90123-1.

36. Handford, P.A., Mayhew, M., Baron, M., Winship, P.R., Campbell, I.D., and Brownlee, G.G. (1991). Key residues involved in calcium-binding motifs in EGF-like domains. Nature *351*, 164–167. https://doi.org/10.1038/351164a0.

37. Mayhew, M., Handford, P., Baron, M., Tse, A.G., Campbell, I.D., and Brownlee, G.G. (1992). Ligand requirements for Ca2+ binding to EGF-like domains. Protein Eng. *5*, 489–494. https://doi.org/10.1093/protein/5.6.489.

38. Rees, D.J., Jones, I.M., Handford, P.A., Walter, S.J., Esnouf, M.P., Smith, K.J., and Brownlee, G.G. (1988). The role of beta-hydroxyaspartate and adjacent carboxylate residues in the first EGF domain of human factor IX. EMBO J. *7*, 2053–2061. https://doi.org/10.1002/j.1460-2075.1988.tb03045.x.

39. Meng, Y., Bo, Z., Feng, X., Yang, X., and Handford, P.A. (2024). The Notch Signaling Pathway: Mechanistic Insights in Health and Disease. Engineering *34*, 212–232. https://doi.org/10.1016/j.eng.2023.11.011.

40. Rand, M.D., Lindblom, A., Carlson, J., Villoutreix, B.O., and Stenflo, J. (1997). Calcium binding to tandem repeats of EGF-like modules. Expression and characterization of the EGF-like modules of human Notch-1 implicated in receptor-ligand interactions. Protein Sci. *6*, 2059–2071.

41. Jensen, S.A., Corbett, A.R., Knott, V., Redfield, C., and Handford, P.A. (2005). Ca2+-dependent Interface Formation in Fibrillin-1. J. Biol. Chem. *280*, 14076–14084. https://doi.org/10.1074/jbc.M412832200.

42. Suk, J.Y., Jensen, S., McGettrick, A., Willis, A.C., Whiteman, P., Redfield, C., and Handford, P.A. (2004). Structural Consequences of Cysteine Substitutions C1977Y and C1977R in Calcium-binding Epidermal Growth Factor-like Domain 30 of Human Fibrillin-1. J. Biol. Chem. *279*, 51258–51265. https://doi.org/10.1074/jbc.M408156200.

43. Palmer, A.G., 3rd (2004). NMR Characterization of the Dynamics of Biomacromolecules. Chem. Rev. *104*, 3623–3640. https://doi.org/10.1021/cr030413t.

44. Braddock, D.T., Cai, M., Baber, J.L., Huang, Y., and Clore, G.M. (2001). Rapid Identification of Medium- to Large-Scale Interdomain Motion in Modular Proteins Using Dipolar Couplings. J. Am. Chem. Soc. *123*, 8634–8635. https://doi.org/10.1021/ja016234f.

45. Chen, K., and Tjandra, N. (2012). The Use of Residual Dipolar Coupling in Studying Proteins by NMR. Top. Curr. Chem. *326*, 47–67. https://doi.org/10.1007/128_2011_215.

46. Fischer, M.W.F., Losonczi, J.A., Weaver, J.L., and Prestegard, J.H. (1999). Domain Orientation and Dynamics in Multidomain Proteins from Residual Dipolar Couplings. Biochemistry *38*, 9013–9022. https://doi.org/10.1021/bi9905213.

47. Prestegard, J.H., Bougault, C.M., and Kishore, A.I. (2004). Residual Dipolar Couplings in Structure Determination of Biomolecules. Chem. Rev. *104*, 3519–3540. https://doi.org/10.1021/cr030419i.

48. Tolman, J.R., and Ruan, K. (2006). NMR Residual Dipolar Couplings as Probes of Biomolecular Dynamics. Chem. Rev. *106*, 1720–1736. https://doi.org/10.1021/cr040429z.

49. Rückert, M., and Otting, G. (2000). Alignment of Biological Macromolecules in Novel Nonionic Liquid Crystalline Media for NMR Experiments. J. Am. Chem. Soc. *122*, 7793–7797. https://doi.org/10.1021/ja001068h.

50. Manalastas-Cantos, K., Konarev, P.V., Hajizadeh, N.R., Kikhney, A.G., Petoukhov, M.V., Molodenskiy, D.S., Panjkovich, A., Mertens, H.D.T., Gruzinov, A., Borges, C., et al. (2021). ATSAS 3.0: expanded functionality and new tools for small-angle scattering data analysis. J. Appl. Crystallogr. *54*, 343–355. https://doi.org/10.1107/S1600576720013412.

51. Ilagan, M.X.G., Lim, S., Fulbright, M., Piwnica-Worms, D., and Kopan, R. (2011). Real-time imaging of notch activation with a luciferase complementation-based reporter. Sci. Signal. *4*, rs7. https://doi.org/10.1126/scisignal.2001656.

52. Whiteman, P., de Madrid, B.H., Taylor, P., Li, D., Heslop, R., Viticheep, N., Tan, J.Z., Shimizu, H., Callaghan, J., Masiero, M., et al. (2013). Molecular basis for Jagged-1/Serrate ligand recognition by the Notch receptor. J. Biol. Chem. *288*, 7305–7312. https://doi.org/10.1074/jbc.M112.428854.

53. Takeuchi, H., Yu, H., Hao, H., Takeuchi, M., Ito, A., Li, H., and Haltiwanger, R.S. (2017). O-Glycosylation modulates the stability of epidermal growth factor-like repeats and thereby regulates Notch trafficking. J. Biol. Chem. *292*, 15964–15973. https://doi.org/10.1074/JBC.M117.800102.

54. Lowenthal, M.S., Davis, K.S., Formolo, T., Kilpatrick, L.E., and Phinney, K.W. (2016). Identification of novel N-glycosylation sites at non-canonical protein consensus motifs. J. Proteome Res. *15*, 2087–2101. https://doi.org/10.1021/acs.jproteome.5b00733.

55. Kettle, S., Yuan, X., Grundy, G., Knott, V., Downing, A.K., and Handford, P.A. (1999). Defective Calcium Binding to Fibrillin-1: Consequence of an N2144S Change for Fibrillin-1 Structure and Function. J. Mol. Biol. *285*, 1277–1287. https://doi.org/10.1006/JMBI.1998.2368.

56. Back, J., Malchiodi, E.L., Cho, S., Scarpellino, L., Schneider, P., Kerzic, M.C., Mariuzza, R.A., and Held, W. (2009). Distinct Conformations of Ly49 Natural Killer Cell Receptors Mediate MHC Class I Recognition in Trans and Cis. Immunity *31*, 598–608. https://doi.org/10.1016/j.immuni.2009.07.007.

57. Wang, Q., Song, H., Cheng, H., Qi, J., Nam, G., Tan, S., Wang, J., Fang, M., Shi, Y., Tian, Z., et al. (2020). Structures of the four Ig-like domain LILRB2 and the four-domain LILRB1 and HLA-G1 complex. Cell. Mol. Immunol. *17*, 966–975. https://doi.org/10.1038/s41423-019-0258-5.

58. Rozbesky, D., Verhagen, M.G., Karia, D., Nagy, G.N., Alvarez, L., Robinson, R.A., Harlos, K., Padilla-Parra, S., Pasterkamp, R.J., and Jones, E.Y. (2020). Structural basis of semaphorin-plexin cis interaction. EMBO J. *39*, e102926. https://doi.org/10.15252/embj.2019102926.

59. Pei, Z., and Baker, N.E. (2008). Competition between Delta and the Abruptex domain of Notch. BMC Dev. Biol. *8*, 4. https://doi.org/10.1186/1471-213X-8-4.

60. Sharma, A., Rangarajan, A., and Dighe, R.R. (2013). Antibodies against the extracellular domain of human Notch1 receptor reveal the critical role of epidermal-growth-factor-like repeats 25–26in ligand binding and receptor activation. Biochem. J. *449*, 519–530. https://doi.org/10.1042/BJ20121153.

61. De Celis, J.F., and Bray, S.J. (2000). The Abruptex domain of Notch regulates negative interactions between Notch, its ligands and Fringe. Development *127*, 1291–1302. https://doi.org/10.1242/dev.127.6.1291.

62. Downing, A.K., Knott, V., Werner, J.M., Cardy, C.M., Campbell, I.D., and Handford, P.A. (1996). Solution structure of a pair of calcium-binding epidermal growth factor-like domains: implications for the Marfan syndrome and other genetic disorders. Cell *85*, 597–605. https://doi.org/10.1016/s0092-8674(00)81259-3.

63. Bork, P., Downing, A.K., Kieffer, B., and Campbell, I.D. (1996). Structure and distribution of modules in extracellular proteins. Q. Rev. Biophys. *29*, 119–167. https://doi.org/10.1017/S0033583500005783.

64. Weisshuhn, P.C., Handford, P.A., and Redfield, C. (2015). 1H, 13C and 15N assignments of EGF domains 4 to 7 of human Notch-1. Biomol. NMR Assign. *9*, 275–279. https://doi.org/10.1007/s12104-014-9591-x.

65. Whiteman, P., Redfield, C., and Handford, P.A. (2014). Bacterial expression and in vitro refolding of limited fragments of the notch receptor and its ligands. Methods Mol. Biol. *1187*, 193–208. https://doi.org/10.1007/978-1-4939-1139-4_15.

66. Liebschner, D., Afonine, P.V., Baker, M.L., Bunkóczi, G., Chen, V.B., Croll, T.I., Hintze, B., Hung, L.-W., Jain, S., McCoy, A.J., et al. (2019). Macromolecular structure determination using X-rays, neutrons and electrons: recent developments in Phenix. Acta Crystallogr. D Struct. Biol. *75*, 861–877. https://doi.org/10.1107/S2059798319011471.

67. Emsley, P., Lohkamp, B., Scott, W.G., and Cowtan, K. (2010). Features and development of Coot. Acta Crystallogr. D Biol. Crystallogr. *66*, 486–501. https://doi.org/10.1107/S0907444910007493.

68. Winter, G. (2010). xia2: an expert system for macromolecular crystallography data reduction. J. Appl. Crystallogr. *43*, 186–190. https://doi.org/10.1107/S0021889809045701.

69. McCoy, A.J., Grosse-Kunstleve, R.W., Adams, P.D., Winn, M.D., Storoni, L.C., and Read, R.J. (2007). Phaser crystallographic software. J. Appl. Crystallogr. *40*, 658–674. https://doi.org/10.1107/S0021889807021206.

70. Jumper, J., Evans, R., Pritzel, A., Green, T., Figurnov, M., Ronneberger, O., Tunyasuvunakool, K., Bates, R., Žídek, A., Potapenko, A., et al. (2021). Highly accurate protein structure prediction with AlphaFold. Nature *596*, 583–589. https://doi.org/10.1038/s41586-021-03819-2.

71. Delaglio, F., Grzesiek, S., Vuister, G.W., Zhu, G., Pfeifer, J., and Bax, A. (1995). NMRPipe: a multidimensional spectral processing system based on UNIX pipes. J. Biomol. NMR *6*, 277–293. https://doi.org/10.1007/bf00197809.

72. Vranken, W.F., Boucher, W., Stevens, T.J., Fogh, R.H., Pajon, A., Llinas, M., Ulrich, E.L., Markley, J.L., Ionides, J., and Laue, E.D. (2005). The CCPN data model for NMR spectroscopy: development of a software pipeline. Proteins *59*, 687–696. https://doi.org/10.1002/prot.20449.

73. Weisshuhn, P.C., Handford, P.A., and Redfield, C. (2015). 1H, 13C and 15N assignments of EGF domains 8–11 of human Notch-1. Biomol. NMR Assign. *9*, 375–379. https://doi.org/10.1007/s12104-015-9613-3.

74. Smallridge, R.S., Whiteman, P., Doering, K., Handford, P.A., and Downing, A.K. (1999). EGF-like domain calcium affinity modulated by N-terminal domain linkage in human fibrillin-1. J. Mol. Biol. *286*, 661–668. https://doi.org/10.1006/jmbi.1998.2536.

75. Kay, L.E., Torchia, D.A., and Bax, A. (1989). Backbone dynamics of proteins as studied by nitrogen-15 inverse detected heteronuclear NMR spectroscopy: application to staphylococcal nuclease. Biochemistry *28*, 8972–8979. https://doi.org/10.1021/bi00449a003.

76. Ottiger, M., Delaglio, F., and Bax, A. (1998). Measurement of *J* and Dipolar Couplings from Simplified Two-Dimensional NMR Spectra. J. Magn. Reson. *131*, 373–378. https://doi.org/10.1006/jmre.1998.1361.

77. Cornilescu, G., Marquardt, J.L., Ottiger, M., and Bax, A. (1998). Validation of Protein Structure from Anisotropic Carbonyl Chemical Shifts in a Dilute Liquid Crystalline Phase. J. Am. Chem. Soc. *120*, 6836–6837. https://doi.org/10.1021/ja9812610.

78. Rambo, R.P., and Tainer, J.A. (2013). Accurate assessment of mass, models and resolution by small-angle scattering. Nature *496*, 477–481. https://doi.org/10.1038/nature12070.

79. Li, J.-L., Sainson, R.C.A., Shi, W., Leek, R., Harrington, L.S., Preusser, M., Biswas, S., Turley, H., Heikamp, E., Hainfellner, J.A., and Harris, A.L. (2007). Delta-like 4 Notch Ligand Regulates Tumor Angiogenesis, Improves Tumor Vascular Function, and Promotes Tumor Growth In vivo. Cancer Res. *67*, 11244–11253. https://doi.org/10.1158/0008-5472.CAN-07-0969.

80. Masiero, M., Li, D., Whiteman, P., Bentley, C., Greig, J., Hassanali, T., Watts, S., Stribbling, S., Yates, J., Bealing, E., et al. (2019). Development of Therapeutic Anti-JAGGED1 Antibodies for Cancer Therapy. Mol. Cancer Therapeut. *18*, 2030–2042. https://doi.org/10.1158/1535-7163.MCT-18-1176.

81. Fiorini, E., Merck, E., Wilson, A., Ferrero, I., Jiang, W., Koch, U., Auderset, F., Laurenti, E., Tacchini-Cottier, F., Pierres, M., et al. (2009). Dynamic Regulation of Notch 1 and Notch 2 Surface Expression during T Cell Development and Activation Revealed by Novel Monoclonal Antibodies1. J. Immunol. *183*, 7212–7222. https://doi.org/10.4049/jimmunol.0902432.

STAR★METHODS

KEY RESOURCES TABLE

REAGENT or RESOURCE	SOURCE	IDENTIFIER
Antibodies		
Rat monoclonal anti-mouse Notch-1 (22E5), APC	Invitrogen	Cat#17-5765-82; RRID: AB_10670632
Bacterial and virus strains		
BL21 pREP4	This paper	N/A
Chemicals, peptides, and recombinant proteins		
Human Notch-1 EGF21-23	This paper	N/A
Human Notch-1 EGF20-23	This paper	N/A
Human Notch-1 EGF20-24	This paper	N/A
Human Notch-1 EGF23-24	This paper	N/A
Human Notch-1 EGF23-24 D909V	This paper	N/A
Human Notch-1 EGF23-27	This paper	N/A
Human Notch-1 EGF23-25	This paper	N/A
Human Notch-1 EGF20-27	This paper	N/A
Drosophila Notch EGF23-24	This paper	N/A
Drosophila Notch EGF23-25	This paper	N/A
Drosophila Notch EGF23-24 D948V	This paper	N/A
Drosophila Notch EGF23-25 D948V	This paper	N/A
Drosophila Notch EGF23-25 N986I	This paper	N/A
^{15}N ammonium chloride	Goss Scientific	NLM-467; Labeled CAS: 2483734-97-15; Unlabeled CAS: 6521-29-12
^{13}C Glucose	Goss Scientific	CLM-1396-5; CAS: 110187-42-3
D_2O	Sigma Aldrich	151882-125G; CAS: 7789-20-0
Deposited data		
BMRB NMR assignments	This paper	BMRB: 51699
Human Notch-1 EGF20-24 structure	This paper	PDB: 9B3N
Human Notch-1 EGF21-23 structure	This paper	PDB: 9B3G
Experimental models: Cell lines		
S2	Expres2ion Biotechnologies, Denmark	Cat#94-005F
HEK293/RBPJk	Ilagan et al.[51]	N/A
HEK293/RBPJk/mN1 WT	Ilagan et al.[51]	N/A
HEK293/RBPJk/mN1 D909V	This paper	N/A
HEK293/RBPJk/mN1 N947I	This paper	N/A
HEK293/RBPJk/mN1 D909V/N947I	This paper	N/A
HEK293/RBPJk/mN1 C993S	This paper	N/A
HEK293/RBPJk/mN1 C960Y	This paper	N/A
HEK293/RBPJk/mN1 S990N	This paper	N/A
HEK293/RBPJk/mN1 L468A	This paper	N/A
B16 WT	Masiero et al.[80]	N/A
B16 mJag1	Masiero et al.[80]	N/A
B16 mDll4	Laboratory of Adrian L. Harris	N/A
Oligonucleotides		
Primers used in this paper, see Table S1.	This paper	N/A

(*Continued on next page*)

Continued

REAGENT or RESOURCE	SOURCE	IDENTIFIER
Recombinant DNA		
Human Notch-1 EGF21-23 in pQE30	This paper	N/A
Human Notch-1 EGF20-23 in pQE30	This paper	N/A
Human Notch-1 EGF23-24 in pQE30	This paper	N/A
Human Notch-1 EGF23-24 D909V in pQE30	This paper	N/A
Human Notch-1 EGF23-27 in pQE30	This paper	N/A
Human Notch-1 EGF20-24 in pEXS2.2	This paper	N/A
Human Notch-1 EGF20-24 in pEXS2.2	This paper	N/A
Human Notch-1 EGF20-27 in pEXS2.2	This paper	N/A
pcDNA5/FRT mN1 L468A	This paper	N/A
pcDNA5/FRT mN1 D909V	This paper	N/A
pcDNA5/FRT mN1 N947I	This paper	N/A
pcDNA5/FRT mN1 D909V/N947I	This paper	N/A
pcDNA5/FRT mN1 C933S	This paper	N/A
pcDNA5/FRT mN1 C960Y	This paper	N/A
pcDNA5/FRT mN1 S990N	This paper	N/A
Drosophila Notch EGF23-24 in pQE30	This paper	N/A
Drosophila Notch EGF23-25 in pQE30	This paper	N/A
Drosophila Notch EGF23-24 D948V in pQE30	This paper	N/A
Drosophila Notch EGF23-25 D948V in pQE30	This paper	N/A
Drosophila Notch EGF23-25 N986I in pQE30	This paper	N/A
Software and algorithms		
Phaser	McCoy et al.[69]	https://phenix-online.org/
Xia2	Winter et al.[68]	https://phenix-online.org/
PHENIX	Liebschner et al.[66]	https://phenix-online.org/
COOT	Emsley et al.[67]	https://www2.mrc-lmb.cam.ac.uk/personal/pemsley/coot/
AlphaFold2	Jumper et al.[70]	https://github.com/google-deepmind/alphafold
CCPN software	Vranken et al.[72]	https://ccpn.ac.uk/
NMRPipe	Delaglio et al.[71]	https://www.ibbr.umd.edu/nmrpipe/
ATSAS package (DAMMIF, DAMAVER, CRYSOL)	Manalastas-Cantos et al.[50]	https://biosaxs.com/software.html
GraphPad Prism 9	GraphPad, San Diego, CA, USA	www.graphpad.com

EXPERIMENTAL MODEL AND STUDY PARTICIPANT DETAILS

Strains used in protein production

The hNotch-1 EGF20-24 and hNotch-1 EGF20-27 protein fragments used in this study were expressed in S2 cells.[18] The hNotch-1 EGF20-23, EGF21-23, EGF23-24, EGF23-25, EGF23-27 protein fragments used in this study were expressed in *Escherichia coli* BL21 cells transformed with a pQE-30 (Qiagen) expression vector and pREP4 plasmid for control of expression via the lac repressor.[30] hNotch-1 EGF23-27 contained serine substitutions at two non-canonical cysteine residues in EGF25 (Cys963Ser) and EGF27 (Cys1040Ser) to facilitate *in vitro* refolding. *Drosophila* Notch fragments EGF23-25, EGF23-25 D948V, EGF23-25 N986I and EGF23-24 were produced using the same system. When cloned into the expression vector, an N-terminal His_6 tag was included for purification, followed by an Ser-Ala spacer and either a factor Xa protease recognition site (Ile-Glu-Gly-Arg) (constructs d/hEGF23–24, d/hEGF23-25, hEGF 23–27) or an enterokinase cleavage site (constructs EGF20-23, EGF21-23) for later removal of the His_6 tag. Ligand constructs were produced as C-terminal Fc and His-tag fusion proteins by transiently transfecting HEK293T cells with cDNA cloned into pHLSec plasmid (hJAG1 NE3)[17] or HEK293F cells with cDNA cloned into pcDNA3.1 (hDLL4 NE3).

CellPress
OPEN ACCESS

METHOD DETAILS

Protein expression, purification, refolding and characterization

Protein expression, isotopic labeling, refolding and purification protocols for preparation of NMR samples have been described previously.[64,65] Briefly for proteins expressed in *E.coli*, cell lysates were spun down at 40,000 rpm for 45 min (Beckman L7–55) and supernatant loaded onto a Ni^{2+} chelating Sepharose column (GE Healthcare). Following elution of His-tagged protein with buffer containing 50 mM EDTA and 100 mM Tris pH 8.3, protein was reduced for 1 h at RT by addition of dithiothreitol (DTT) to a final concentration of 0.1 M. The pH was adjusted to pH ~2, by addition of concentrated HCl, and the solution was dialyzed against 0.1% (v/v) trifluoroacetic acid (TFA) overnight. Following filtration (0.2 μM filter (Millex-GP)), the soluble fraction was purified by reverse-phase HPLC (C8 column) using a Beckman Gold system. Purified, reduced protein was subsequently refolded in an oxido-shuffling buffer containing 100 mM Tris–HCl pH 8.3, 3 mM L-cysteine, 0.3 mM L-cystine, 0.2 mg/mL protein at 37°C for ~48 h. Following acidification to pH ~2, dialysis was performed against 0.1% (v/v) TFA for >5 h. Protein was subsequently concentrated by ultrafiltration and purified by HPLC. After lyophilization, the His_6 tag was removed by incubation overnight with either one unit of bovine factor Xa (DENZYME) per mg of protein in 50 mM Tris–HCl pH 7.5, 0.1 M NaCl, 1 mM $CaCl_2$ at 37°C (or enterokinase (NEB) in same buffer) were incubated with an enzyme:protein ratio by weight of 1:1,000,000 overnight at RT. Protein was further purified by cation-exchange fast liquid protein chromatography and HPLC. hNotch-1 receptor constructs EGF20-24 and EGF20-27 were recombinantly expressed in S2 insect cells (Expres2ion Biotechnologies, Denmark) as C-terminal His-tagged fusion proteins.[18] Medium containing recombinantly expressed protein was filtered and loaded onto a cOmplete His-tag Purification Column (Roche Diagnostics, UK), for purification. Following washing with 50 mM Tris pH 9.0, 2.5 mM imidazole, 200 mM NaCl and 5 mM $CaCl_2$, proteins were eluted with wash buffer containing 250 mM imidazole. Proteins were further purified by size-exclusion chromatography (SEC) using a Superdex S200 (GE Healthcare, Sweden) preparative column in 10 mM Tris pH 7.5, 200 mM NaCl and 5 mM $CaCl_2$ buffer. Each nanomole of protein was incubated with 0.5 units of N-terminal His-tagged HRV-3C protease (Sigma) overnight at 4°C for His tag removal. Non-cleaved protein and HRV-3C protease were removed through incubation with Ni-NTA agarose (Qiagen) for 1 h at 4°C. The supernatant was collected and purified by SEC. Fc and His-tagged ligand proteins for activation assays were purified as previously described.[17] Cell medium containing hJAG1 NE3 protein was mixed with 3 times volumes of 50 mM Tris and 200 mM NaCl (pH 9.0). Cell medium containing hDLL4 NE3 was mixed with 2 times volumes of 20 mM MES and 200 mM NaCl (pH 6.5). The diluted medium was filtered and loaded onto a cOmplete His-tag Purification Column (Roche Diagnostics, UK) for purification. Following washing with 50 mM Tris, 10 mM imidazole and 200 mM NaCl (pH 9.0) for hJAG1 NE3 or 20 mM MES, 50 mM imidazole and 200 mM NaCl (pH 6.5) for hDLL4 NE3, proteins were eluted with wash buffer containing 300 mM imidazole. Proteins were further purified by SEC using a SuperdexTM S200 (GE Healthcare, Sweden) preparative column in 50 mM Tris and 200 mM NaCl (pH 7.5).

X-Ray crystallography

Human Notch-1 EGF21-23 was crystallized by vapor diffusion from sitting drops at 9 mg/mL, with 100 nL mother liquor (0.2 mM ammonium acetate, 0.1 M BIS-Tris pH 5.5, 25% (w/v) PEG3350) and 100 nL protein solution (25 mM Tris pH 7.5, 150 mM NaCl, 10 mM $BaCl_2$). Crystals were cryo-protected by addition of 30% (v/v) ethylene glycol, and data collected at Diamond Light Source on beamline I04-1. The dataset was indexed, integrated and scaled using Xia2 (Winter, 2010). The structure was phased and built iteratively using AutoSol in PHENIX (dev-4694),[66] using the anomalous signal from Ba^{2+}. The structure was refined using PHENIX.refine (dev-4694),[66] with COOT used for manual rebuilding and inspection.[67]

Human Notch-1 EGF20-24 was crystallized by vapor diffusion from sitting drops at 20.7 mg/mL, with 200 nL mother liquor (0.2 mM imidazole malate pH 6, 8% (w/v) PEG4K) and 200 nL protein solution (5 mM Tris pH 7.5, 50 mM NaCl, 10 mM $CaCl_2$). Crystals were cryo-protected by addition of 30% (v/v) ethylene glycol and 40 mM $CaCl_2$, and data collected at Diamond Light Source on beamline I03. The dataset was indexed, integrated and scaled using Xia2.[68] The structure was phased using molecular replacement in Phaser,[69] using the hN-1 EGF21-23 and AlphaFold2[70] models for EGFs 20 and 24. The structure was refined using PHENIX.refine (dev-4694),[66] with COOT used for manual rebuilding and inspection.[67]

NMR spectroscopy

All NMR experiments were carried out using spectrometers operating at ^1H frequencies of 500, 600 and 750 MHz equipped with Bruker Avance II (500 MHz) or Avance IIIHD (600 and 750 MHz) consoles and 5mm TCI cryoprobes. Data were processed using NMRPipe[71] and spectra were analyzed using the CCPN software.[72]

Resonance assignments for EGF20-23 were carried out using $^{13}C/^{15}N$ labeled protein and standard methods as described previously.[64,73] Unless otherwise stated, all NMR experiments were carried out at 25°C in 5 mM Tris-HCl, 150 mM NaCl at pH 7.5 in 95% H_2O/5% D_2O.

Ca^{2+} dissociation constants for the hNotch-1 EGF20-23 and EGF23-24 constructs were obtained from Ca^{2+} titrations monitored by 2D ^1H-^{15}N HSQC experiments, collected at 500 MHz. Values for EGF23-25, *Drosophila* Notch dN EGF23-24 and dN EGF23–25 were obtained from 1D and 2D ^1H NMR experiments.[41,42,74] Samples were initially Ca^{2+} free and the Ca^{2+} concentration was increased by addition of $CaCl_2$ aliquots up to saturating concentrations (usually ~25 mM).

{^1H}-^{15}N heteronuclear NOE experiments were carried out on ^{15}N-labeled EGF20-23 in order to examine the sub-nanosecond dynamics of specific amides.[75] Samples for measurement of the heteronuclear NOE contained 800μM EGF20-23 and 1.4 mM, 2.8 mM

or 40mM $CaCl_2$. The samples with 1.4 mM and 2.8 mM $CaCl_2$ contained 150 mM NaCl while the 40 mM $CaCl_2$ did not. With 1.4 mM $CaCl_2$, the EGF23 site will be fully occupied with Ca^{2+} while EGF21 and EGF20 will be ~23% and ~4% occupied, respectively. With 2.8 mM $CaCl_2$, the EGF23 site will be fully occupied with Ca^{2+} while EGF21 and EGF20 will be ~55% and ~14% occupied, respectively. Spectra with and without ^{1}H saturation were collected as interleaved experiments; the {^{1}H}-^{15}N NOE was calculated as the ratio of the peak intensities in the spectra recorded with and without ^{1}H saturation. Data were collected at a ^{1}H frequency of 750 MHz. ^{1}H saturation was applied for 4 s.

Residual dipolar couplings (RDCs) were collected for the EGF20-23 using liquid crystalline media containing n-alkyl-poly(ethylene glycols) (PEG) and *n*-alkyl alcohols as described previously.[49] The final concentration of C12E6/hexanol used was 2%. EGF20-23 samples for measurement of the RDCs contained 540 μM EGF20-23 and 0.84 mM or 40 mM $CaCl_2$. The sample with 0.84 mM $CaCl_2$ contained 150 mM NaCl while the 40 mM $CaCl_2$ sample did not. With 0.84 mM $CaCl_2$, the EGF23 site will be fully occupied with Ca^{2+} while EGF21 and EGF20 will be ~15% and ~2% occupied, respectively. With 40 mM $CaCl_2$, the EGF 21 and EGF23 sites will be fully occupied with Ca^{2+} while EGF20 will be ~75% occupied. Isotropic spectra and aligned spectra were collected for protein solutions in 90% H_2O/10% D_2O using the interleaved IPAP experiment[76] performed at a ^{1}H frequency of 500 MHz. Residual dipolar couplings were measured as the difference between the splitting observed in the isotropic and aligned datasets. RDC values for EGF20, EGF21, EGF22 and EGF23 were fitted to the X-ray coordinates of EGF20-24, using an in-house program. The overall fit between experimental and calculated RDC values was assessed using the quality factor (Q) defined as: $Q = [\sum_{i=1,\ \ldots,N} (RDC^{expt} - RDC^{calc})^2/N]^{½}/RDC_{rms}$.[77] With 0.84 mM $CaCl_2$, Q values of 0.24, 0.23, 0.23 and 0.21 were obtained for EGF20, EGF21, EGF22 and EGF23. With 40 mM $CaCl_2$, Q values of 0.23, 0.22, 0.24 and 0.21 were obtained for EGF20, EGF21, EGF22 and EGF23. These Q values indicate a good fit of the experimental RDCs to the X-ray structure.

SAXS

Data collection on hNotch-1 EGF23-27 and EGF20-27 samples was carried out at the Diamond Light Source at Beamline B21 using a SEC-SAXS method. 100 μL samples were prepared (100 μL, 2.5 mg/mL, 5 mM Tris pH7.5, 15 mM $CaCl_2$), including dialysis in 2 L of buffer overnight at 4°C (Table 3). Samples were subsequently frozen at −80°C for transport to the beamline and thawed immediately prior to use. SAXS samples (50 μL) were autoloaded onto a Shodex 402.5 analytical SEC column. The eluent from this column then passed through the X-ray beam and scatter pattern recorded. Data were collected up to a scattering vector (s) of 0.37 $Å^{-1}$. Scatter resulting from buffer was subtracted from the peak corresponding to protein scatter using the program SCATTER 3.0[78] and the ATSAS software package[50] was used for further analysis. Guinier analysis and *ab initio* modeling and structure fitting was carried out using the program SREFLEX in the ATSAS software package.[50] Modeling was performed at default settings using 10 repetitions followed by DAMAVER. CRYSOL was subsequently used to confirm the data fits the structure model. A model for hNotch-1 EGF20-27 was also generated using AlphaFold2.[70]

HEK-RBP cells growth and transfection

mN1 Ax variants were created by overlap extension PCRs[41] using a cDNA fragment from 521 bp to 4131 bp of the full-length mN1. The mutated cDNA fragment was initially inserted between the SbfI and BamHI sites of plasmid pUC19 and following sequencing and amplification, was digested and ligated into pcDNA5/FRT plasmid containing the remainder of the mN1 cDNA.

HEK-RBP cells were maintained in DMEM medium, supplemented with 10% FCS (Gibco), 2 mM L-glutamine (Gibco), 50 U/mL penicillin, 0.05 mg/mL streptomycin (Sigma) and 0.2 μg/mL puromycin. Cells were transfected using Mirus TransIT-293 Transfection Reagent and carried out using 80% confluent wells in a 6-well plate, using a total of 1000 ng of DNA per transfection (pOG44: pcDNA5/FRT mN1 at a ratio of 9:1). 24 h following transfection, cells were transferred to 15 cm dishes and additional selection (100 μg/mL hygromycin) applied. Clones were subsequently transferred to 6-well plates and expanded in the presence of both 0.2 μg/mL puromycin +100 μg/mL hygromycin.

B16 cells growth conditions

B16 cells expressing either mDll4 or mJag1 were maintained in RPMI medium, supplemented with 10% FCS (Gibco), 50 U/mL penicillin and 0.05 mg/mL streptomycin (Sigma) and split 1:15 once confluent.[79,80]

Notch activation assays

Ligand-independent assay

Established HEK-RBP cell lines expressing Notch-1 variants were split 1:3 into DMEM +10% FCS. After two days, 40,000 cells were added to each well of a 96-well Costar black clear bottom plate (DMEM +10% FCS). 48 h later, wells were washed with HBS (20 mM HEPES pH 7.4, 150 mM NaCl, 0.9 mM Ca^{2+}), and cells resuspended in 100 μL HBS +150 μg/mL D-luciferin +10 mM EGTA. Control wells were treated with substrate solution without EGTA. Luciferase activity was monitored using a BMG LABTECH PHERAstar FS plate reader at 40 min after addition of EGTA.

Ligand-dependent assay

As for ligand independent but 96-well plates were pre-coated with 50 μL of 10 μg/mL purified hJAG1 NE3 or hDLL4 NE3 in HBS overnight at 4°C, control wells were incubated without hJAG1 or hDLL4. Subsequently wells were washed with HBS, and reporter cells added. 24 h later wells were washed with HBS, and cells resuspended in 100 μL substrate solution added (150 μg/mL D-luciferin in HBS +1 mg/mL Mg^{2+}, 0.1% glucose). Luciferase activity was measured immediately. For co-culture assays: B16 ligand expressing

cells (either mJag1 or mDll4) were split 1:15 into RPMI +10% FCS. 48 h later, 40,000 Notch-1 expressing cells were added to each well of a white 96-well Nunc MicroWell plate (DMEM +10% FCS) and grown overnight. An equivalent number of B16 cells were added to each well and 24 h later, wells were washed with HBS, 100 μL substrate solution added and luciferase activity measured immediately.

Cis-inhibition assay

Established HEK-RBP cell lines expressing Notch-1 variants were split into 6-well plates or T25 cell culture flasks and grown to 70–80% confluency. Cells were transiently transfected with pcDNA3.1 full-length hJAG1 or pcDNA3.1 empty vector using Mirus TransIT-293 Transfection Reagent. The presence of hJAG1 in the same cell (*cis*) as the Notch WT/variant provides *cis*-inhibitory potential in these cells. 24 h after transient transfection, 40,000 cells in DMEM +10% FCS were transferred to each well and incubated overnight. An equivalent number of B16 cells were then added to each well and, after 24 h, wells were subsequently washed with HBS, and luciferase activity measured as described above.

Flow cytometry-based cell surface Notch-1 quantification

5×10^5 cells expressing each Notch variant were harvested in a microcentrifuge tube and washed 2 times in 400 μL of FACS buffer (PBS, 0.1% W/V NaN_3, 0.2% V/V FBS, sterile-filtered). Washed cells were incubated for 1 h in the dark in 100 μL FACS buffer with 2 μg/mL allophycocyanin (APC)-conjugated anti-mNotch-1 (22E5) antibody (Invitrogen #17-5765-82, initially created by Fiorini et al.[81]). Cells were washed 2 times with FACS buffer, resuspended in 400 μL PBS with 1 μg/mL DAPI and incubated for 10–15 min before flow cytometry analysis. All staining steps were performed in the dark with prechilled buffer solutions on ice or at 4°C. Samples were analyzed with a CytoFLEX LX flow cytometer (Beckman Coulter) with a flow rate between 200 and 1000 events/second. Data for 30,000 events were collected for each sample. DAPI fluorescence was measured with excitation at 405 nm and emission at 450 nm. APC fluorescence was measured with excitation at 638 nm and emission at 660 nm. Data were analyzed by FlowJo v10.8 Software (BD Life Sciences). For each sample, the median fluorescence intensity of APC for viable single cells was quantified and used to show the median cell surface Notch-1 level. Four biological repeats were performed for each cell line.

QUANTIFICATION AND STATISTICAL ANALYSIS

X-ray crustallography data collection and refinement statistics are shown in Table 1. SAXS data collection statistics are shown in Table 3. Parametric and nonparametric tests used are indicated in each figure legend, together with (where appropriate) the post-hoc test used for multiple comparisons. Values are presented together with the mean ± SD (standard deviation). ns = not significant; *$p < 0.05$, **$p < 0.01$, ***$p < 0.001$, ****$p < 0.0001$; Statistical data were analyzed with Prism 9 (GraphPad, San Diego, CA, USA). SD is calculated using the following equation:

$$SD = \sqrt{\frac{\Sigma(x - \bar{x})^2}{n - 1}}$$

Structure, Volume *32*

Supplemental Information

Structural and functional studies of the EGF20-27 region reveal new features of the human Notch receptor important for optimal activation

Zhihan Bo, Thomas Rowntree, Steven Johnson, Hilman Nurmahdi, Richard J. Suckling, Johan Hill, Boguslawa Korona, Philip C. Weisshuhn, Devon Sheppard, Yao Meng, Shaoyan Liang, Edward D. Lowe, Susan M. Lea, Christina Redfield, and Penny A. Handford

Supplemental Information

Structural and functional studies of the EGF20-27 region reveal new features of the Notch receptor important for optimal activation.

Zhihan Bo[1‡], Thomas Rowntree[1‡], Steven Johnson[2#‡], Hilman Nurmahdi[1], Richard Suckling[2], Johan Hill[1], Bogusia Korona[1], Philip C. Weisshuhn[1], Devon Sheppard[2], Yao Meng[1], Shaoyan Liang[1], Edward D. Lowe[1], Susan M. Lea[2#*], Christina Redfield[1,*] & Penny A. Handford[1,*]

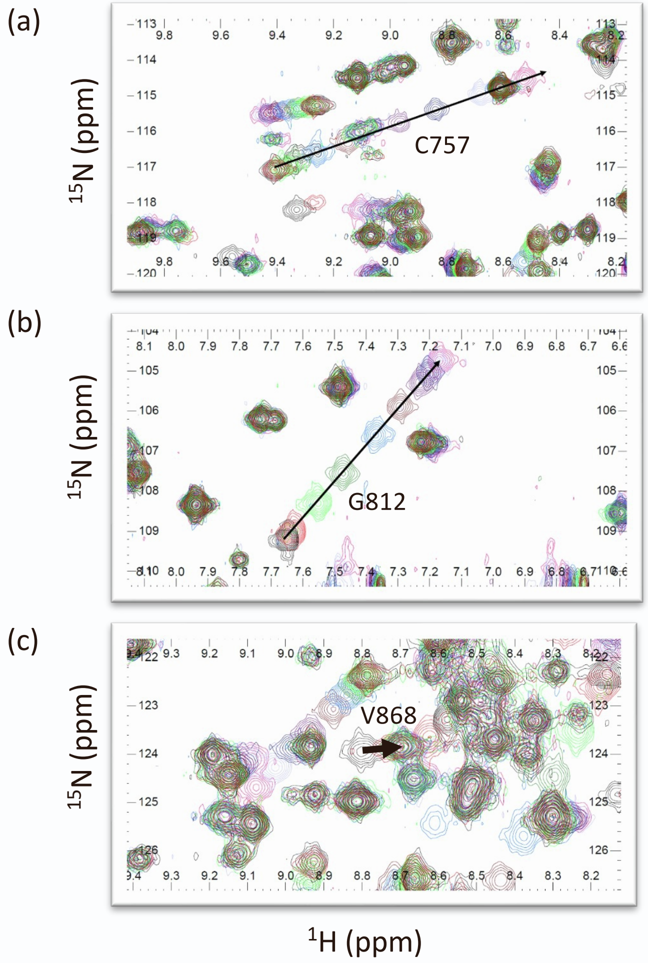

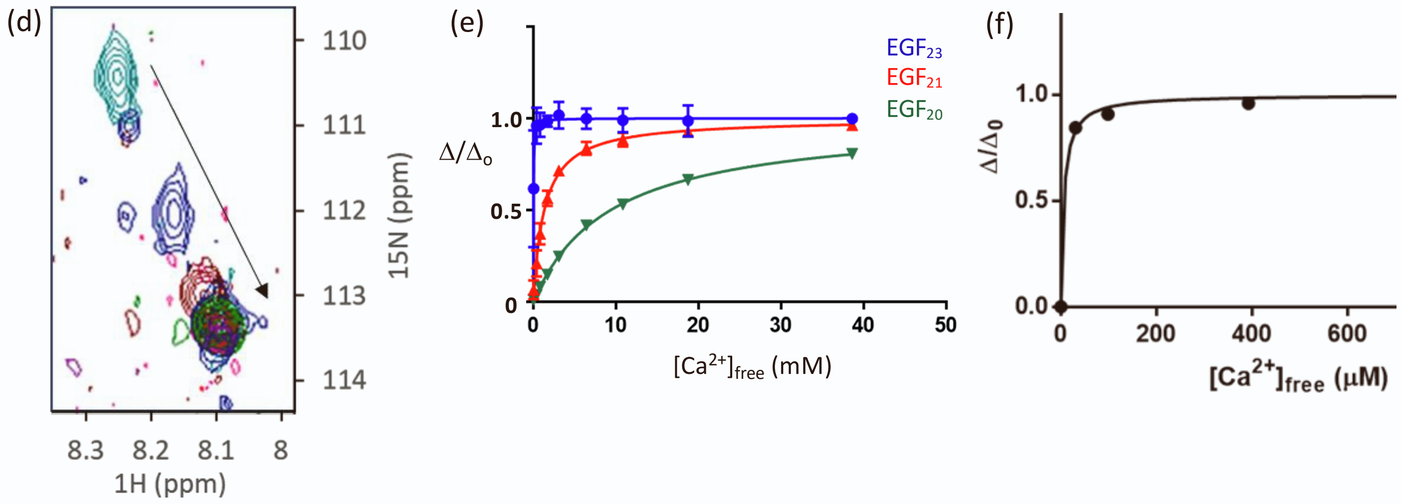

Figure S1 Determination of Ca^{2+} K_D values for Ca^{2+}-binding domains of hNotch-1 (EGF20, EGF21, EGF23 and EGF24) from ^1H-^{15}N HSQC spectra collected at 500 MHz for EGF20-23 and EGF23-24. Related to Figure 1.

(a)-(c) Overlays of zoomed in regions of HSQC spectra of EGF20-23 collected at 298K for Ca^{2+} concentrations of 0, 0.5, 1.0, 1.5, 2.5, 4.0, 7.5, 12.0, 20.0 and 40.0 mM showing Ca^{2+} dependent chemical shift changes. (a) The arrow indicates how the C757 peak in EGF20 shifts with increasing $[Ca^{2+}]$. (b) The arrow indicates how the G812 peak in EGF21 shifts with increasing $[Ca^{2+}]$. (c) The arrow indicates how the V868 peak in EGF23 shifts with increasing $[Ca^{2+}]$. (d) Overlay of zoomed in regions of HSQC spectra of EGF23-24 collected at 298K for Ca^{2+} concentrations of 0, 0.1, 0.2 and 0.6 mM showing Ca^{2+} dependent chemical shift changes. The arrow indicates how a peak arising from a glycine residue in EGF24 shifts with increasing $[Ca^{2+}]$. (e)-(f) Fractional changes (Δ/Δ_o) in the combined HN/^{15}N chemical shifts for residues in EGF20, EGF21 and EGF23 (e) and in EGF24 (f) are plotted against the concentration of free Ca^{2+} to yield K_D values of 9mM, 1.3mM, 23μM and <25μM for EGF20, EGF21, EGF23 and EGF24, respectively. Chemical shift changes were fitted to the equation $\Delta/\Delta_o = [Ca^{2+}]_{free}/([Ca^{2+}]_{free} + K_D)$, where Δ is the observed chemical shift change at each value of $[Ca^{2+}]_{free}$ and Δ_o is the maximum observed chemical shift change. Error bars shown for Δ/Δ_o represent estimated errors involved in measuring chemical shift values.

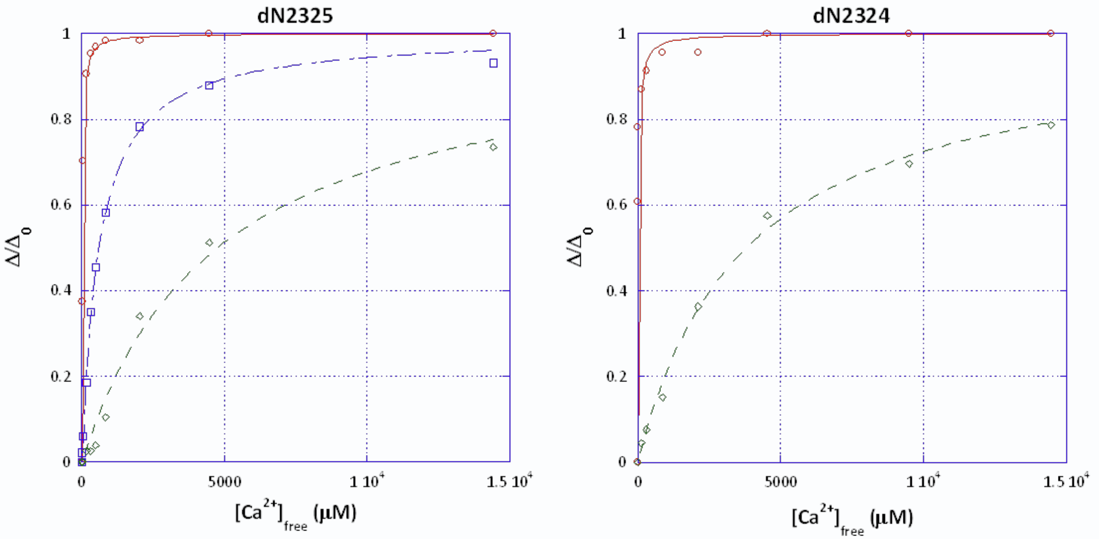

Figure S2. Quantification of Ca^{2+}-binding sites in Drosophila Notch (dN) EGF23-25, EGF23-24 by 2D-NOESY. Related to Figure 1.

Plots of Ca^{2+}-dependent chemical shift changes in EGF23, 24, and 25 are coloured green, red and blue respectively. K_D values of each domain in dN EGF23-25 were ~5mM, ~20μM and~600μM respectively. K_D values of EGF23 and 24 in dN EGF23-24 were ~4mM and ~20μM respectively.

Figure S3. Quantification of Ca^{2+}-binding sites in Drosophila Notch (dN) EGF23-25, EGF23-24, and Abruptex variants EGF23-25_948 (D948V in EGF24), and EGF23-25_986 (N986I in EGF25) by 2D-NOESY. Related to Figure 1.

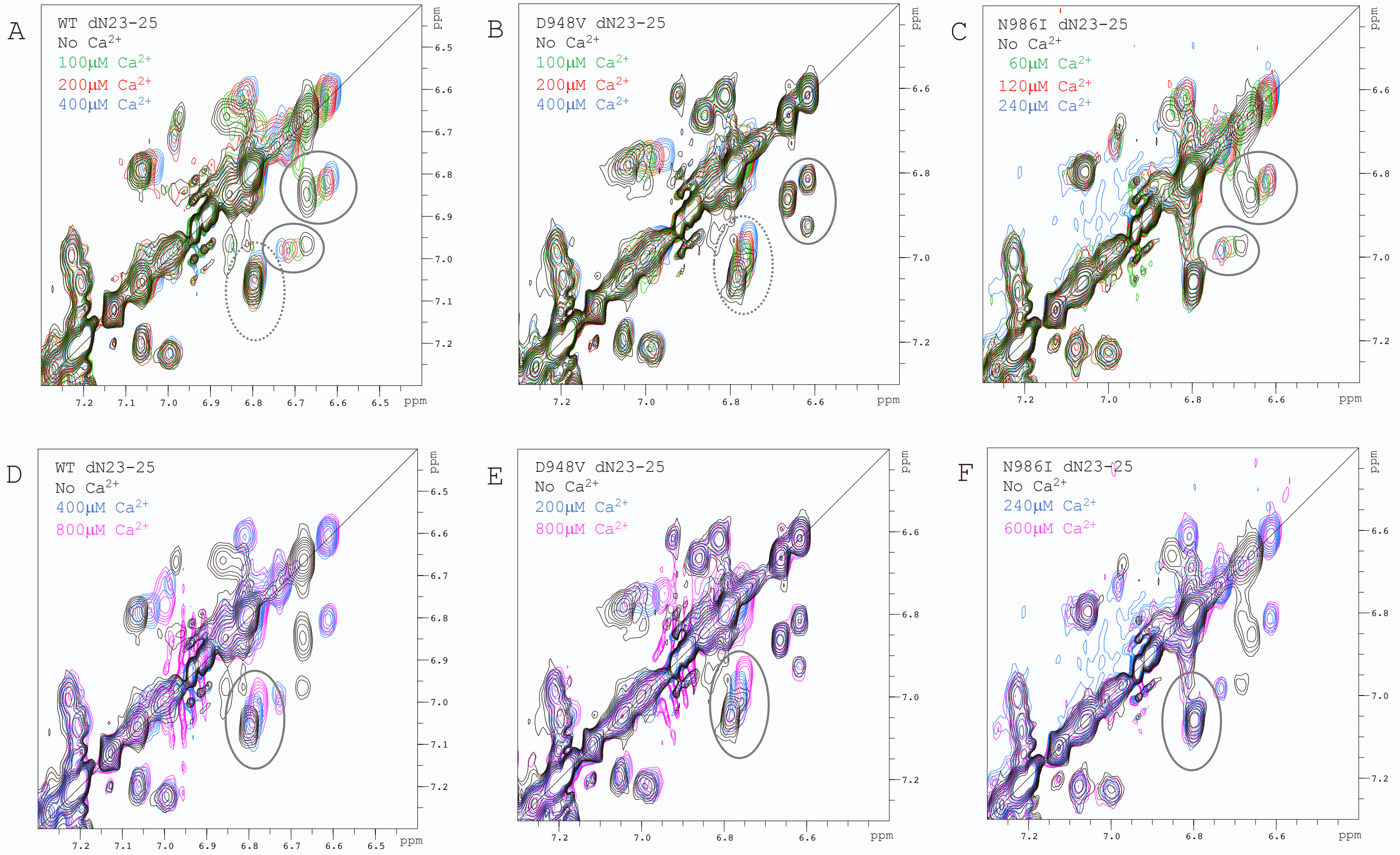

Figure S3 legend. Quantification of Ca^{2+}-binding sites in Drosophila Notch (dN) EGF23-25, EGF23-24, and Abruptex variants EGF23-25_948 (D948V in EGF24), and EGF23-25_986 (N986I in EGF25) by 2D-NOESY. Related to Figure 1.

2D ^1H-^1H NOESY spectra for WT EGF23-25 and the two variants at various [Ca^{2+}] were used to characterise Ca^{2+} binding in EGF24 and EGF25. In A, peaks from aromatic residues assigned to EGF24 (solid gray ovals) shift at Ca^{2+} concentrations between 0 and 400 μM indicating a relatively high affinity binding site. The concentration of the WT EGF23-25 was 280μM. These peaks were assigned to EGF24 by comparison with spectra for EGF23-24. In B, these peaks do not move indicating that the D948V substitution in EGF24 greatly reduces the affinity for Ca^{2+} in EGF24. In C, these peaks shift in a manner similar to that in WT EGF23-25 indicating that the N986I substitution in EGF25 does not affect the affinity for Ca^{2+} in EGF24. In D, a peak from an aromatic residue assigned to EGF25 (solid gray oval) shifts at Ca^{2+} concentrations between 0 and 800 μM. This peak is highlighted in A by the dotted oval. Comparison of A and D shows that this peak only shifts at higher Ca^{2+} concentrations, indicating a weaker affinity binding site in EGF25 compared to EGF24. This peak was assigned to EGF25 by comparison with spectra for EGF23-24, where it is absent. In E, this peak shifts in a manner similar to that seen in D, indicating that the D948V substitution in EGF24 does not affect the affinity for Ca^{2+} in EGF25. In F, this peak does not move indicating that the N986I substitution in EGF25 greatly reduces the affinity for Ca^{2+} in EGF25.

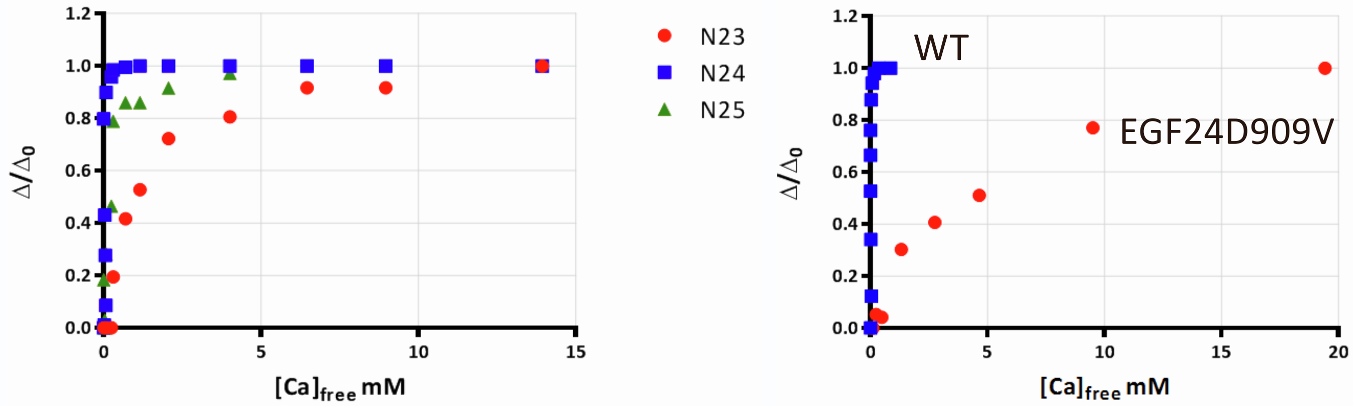

Figure S4. Analysis of homologous Abruptex missense mutation D909V in hNotch-1 EGF24. Related to Figure 1.

hNotch-1 23-25 has 3 calcium-binding sites. D909V reduces the affinity of EGF24 site by 50-100x

Figure S5 SAXS data for hNotch-1 EGF23-27 compared to EGF20-27 . Related to Figure 4.

A scaled, merged and averaged X-ray scattering curve collected with purified hNotch-1 EGF23-27 at 2.5 mg/ml in 5 mM Tris, pH 7.5, 15 mM $CaCl_2$. Data are shown compared to EGF20-27. The P(r) distribution shows the expected difference in length for the two fragments comprising 5 and 8 EGF domains respectively.

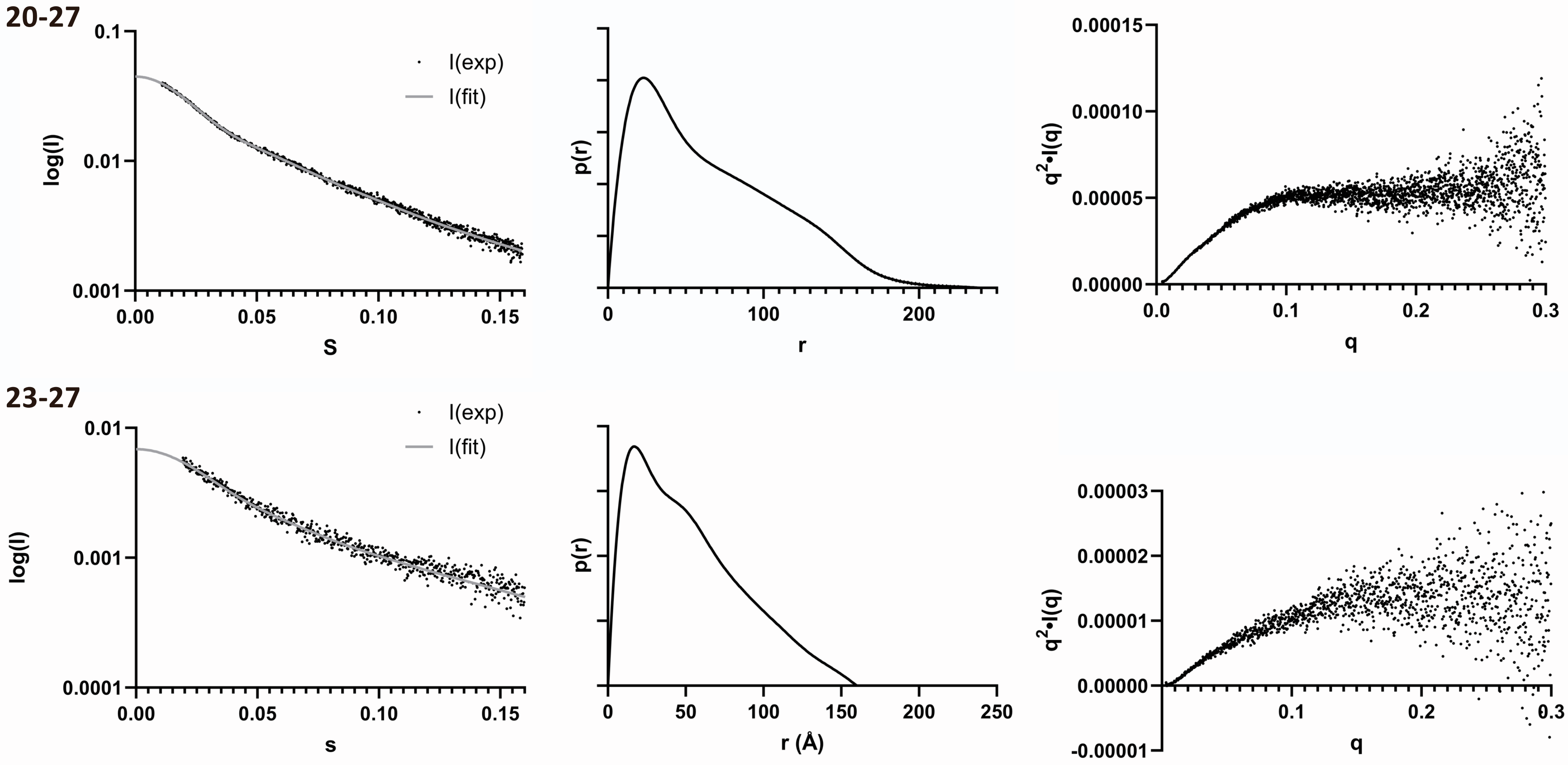

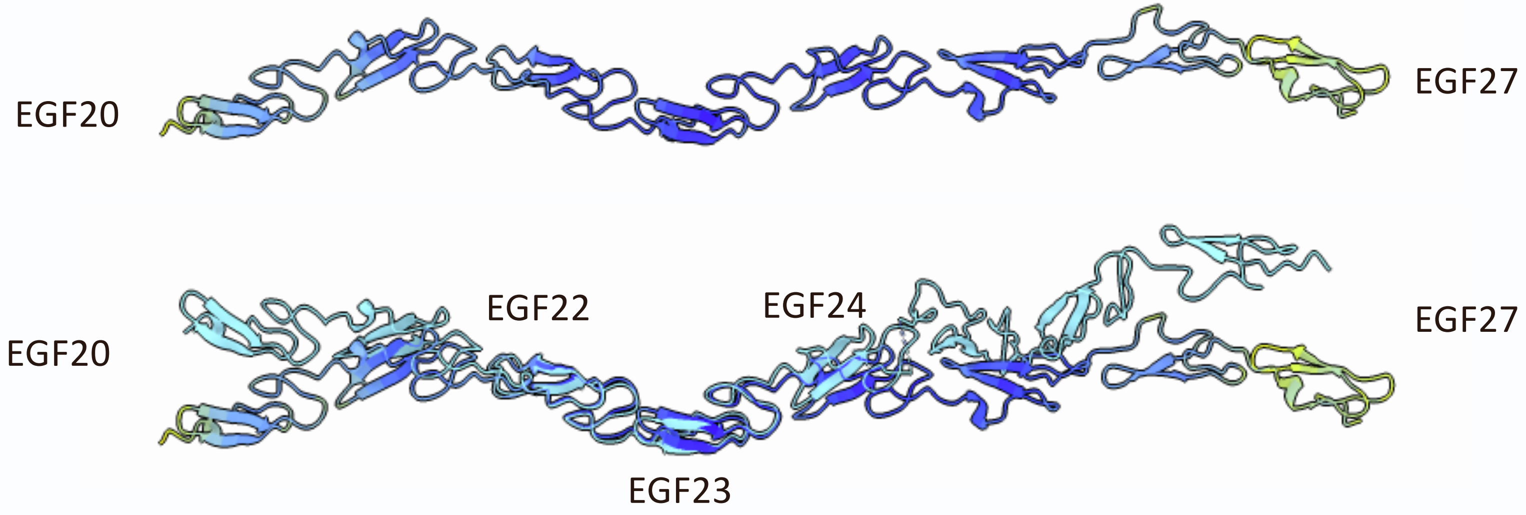

Figure S6. Comparison of AlphaFold2 model for EGF20-27 with the model derived from the SAXS data. Related to Figure 4.

Top: Cartoon representation of the highest ranked AlphaFold2 model generated for EGF20-27. Colouring is based on the pLDDT confidence score (high confidence in dark blue, low confidence in yellow).

Bottom: The AlphaFold2 model is aligned with the model generated by SREFLEX using the SAXS data.

The AlphaFold2 colouring is the same as in the top figure while the SREFLEX structure is shown in light blue.

The two models overlay well for domains EGF22-24 but deviate at both the N- and C-termini. Although both models show an elongated structure, the AlphaFold2 model appears to be slightly more extended. The SREFLEX model gives better agreement with the SAXS data ($\chi 2$ = 1.96) than the AlphaFold model ($\chi 2$ = 3.35), as assessed by CRYSOL.

Figure S7. Luciferase reporter assays of Notch activity. Related to Figure 5.

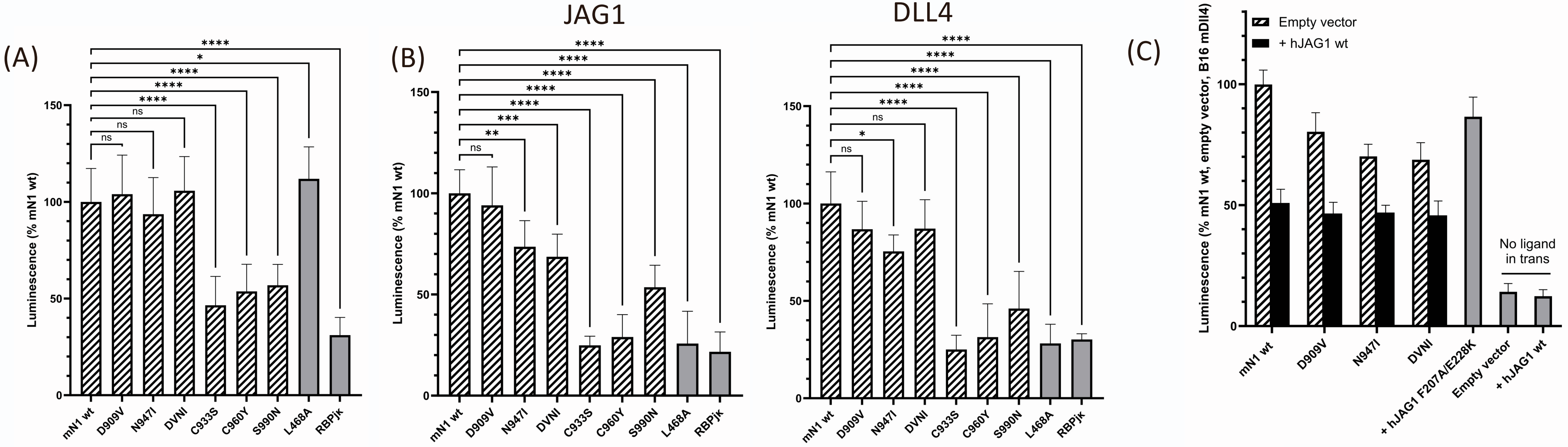

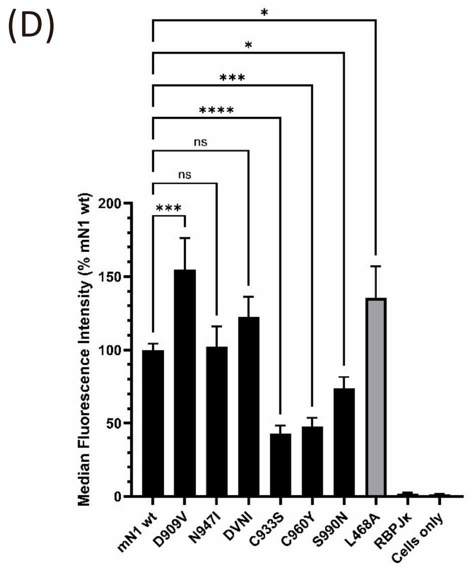

(A) Ligand-independent activation: Graphs show combined results of four independent plates, each with five repeat wells. The signal in each case is shown relative to Notch-1 wt (mN1 wt). Data presented as mean ± SD. Statistical significance was determined by ordinary one-way ANOVA and Dunnett post-hoc test (ns= no significance, * = P < 0.05, ** = P < 0.01, *** = P < 0.001, **** = P < 0.0001).

(B) Ligand-dependent transactivation using purified NE3 ligand constructs: Graphs show the combined results of four independent plates, each with five repeat wells. The signal in each case is shown relative to Notch-1 wt (mN1 wt). Data presented as mean ± SD. Statistical significance was determined by Kruskal-Wallis test and Dunn's post hoc test (ns= no significance, * = P < 0.05, ** = P < 0.01, *** = P < 0.001, **** = P < 0.0001).

(C) Cis-inhibition of Notch variants co-cultured with B16 cells expressing mDll4: Graphs show combined results of 4 independent plates, each with five repeat wells. Signal in each case is shown relative to Notch-1 wt (mN1 wt) transfected with empty pcDNA3.1, co-cultured with B16 mDll4 expressing cells. Data presented as mean ± SD.

(D) Direct measurement of cell surface Notch variants by FACS: Data presented as mean ± SD. Statistical significance was determined by Kruskal-Wallis test and Dunn's post hoc test (ns = not significant, * p<0.05, ** p<0.01, *** p<0.001, **** p < 0.0001).

Figure S8. Activity data showing full-length JAG1 variant F207A/E228K has a reduced ability to transactivate WT Notch-1, as well as a reduced ability to cis-inhibit, suggesting the ligand DSL domain -Notch LBR interaction may be common to both modes of regulation. Related to Figure 5.

Luciferase reporter assay shows JAG1 F207A/E228K variant has a reduced ability to trans-activate Notch-1. Notch-1 wt cell line co-cultured with cells transfected with pcDNA3.1 full-length hJAG1 wt, pcDNA3.1 hJAG1F207A/E228K, or empty pcDNA3.1 vector. Graphs show the combined results of four independent plates, each with five repeat wells. The signal in each case is shown relative to JAG1 wt. Data presented as mean ± SD. Statistical significance was determined by Mann-Whitney test (**** = $P < 0.0001$).

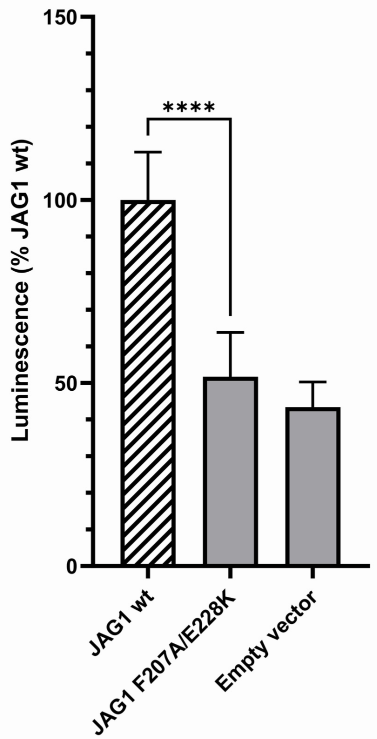

Table S1. Primers used for cloning and mutagenesis in this study. Primers were purchased from Thermo Fisher Scientific. Related to STAR Methods.

Primer	Sequence (5' - 3')
pcDNA5/FRT mN-1 mutagenesis	
For_end_Sbf1	ACACCTGCAGGCAAGATGTTAATGAGTGCAGCCAGAACCC
Rev_end_BamH1	GAAGGATCCCAGGCATAGGCAGGTGGGACTACGTGGG
mouseN1_L468A_F	CAGAATGATGCCACTTGCGCCGACCAGATTGGGGAGTTC
mouseN1_L468A_R	GAACTCCCCAATCTGGTCGGCGCAAGTGGCATCATTCTG
mouseN1_D909V_F	AACTGTGAGAGTGACATCGTGGACTGCCGCCCCAACCCG
mouseN1_D909V_R	CGGGTTGGGGCGGCAGTCCACGATGTCACTCTCACAGTT
mouseN1_C933S_F	CTTCTGCGACAGTCTGCCCGGCTTCCAGGGTG
mouseN1_C933S_R	CCGGGCAGACTGTCGCAGAAGGCTGTGTTGATG
mouseN1_N947I_F	TTCTGTGAGGAGGACATCATTGAATGTGCCAGCAATCCC
mouseN1_N947I_R	GGGATTGCTGGCACATTCAATGATGTCCTCCTCACAGAA
mouseN1_C960Y_F_2	CCAAAATGGTGCCAATTATACTGACTGTGTGGACAGCTACAC
mouseN1_C960Y_R_2	GCTGTCCACACAGTCAGTATAATTGGCACCATTTTGGCAGGG
mouseN1_S990N_F_2	CACCTGACTGTACTGAGAATTCCTGCTTCAATGGTGGTACCTG
mouseN1_S990N_R_2	CCACCATTGAAGCAGGAATTCTCAGTACAGTCAGGTGTGTTG
pQE30 hNotch-1 EGF21-23 cloning	
EGF21EK_F	TAGTAGGGATCCGATGACGATGACAAATCAGCAACCAACATCAACG AGTGTGCGTC
EGF23_R	TAGTAGAAGCTTCTATTAGGTCTCGCAGTTGCGCCCACTG
pEXS2.2 hNotch-1 EGF20-24 cloning	
F4_N110-13	TTAAGAATTCGCCACCATGAAGCTGTGCATC
AbiN1-24R	ATAGGATCCCTCCTCGCAGAAGGTGCCGCG
pEXS2.2 hNotch-1 EGF20-27 cloning	
F4_N110-13	TTAAGAATTCGCCACCATGAAGCTGTGCATC
AbiN1-27R	ATAGGATCCGTTCTGGCAATTCGGACCGGTG
pQE30 hNotch-1 EGF23-27 cloning	
23-27CSCS_F	TAGTAGAGAGATCTATAGAAGGACGATCAGCAGTCGACATCAACGA GTGCG
23-27CSCS_R	CACTGGCCCCAACTGCCAGCTCGAGTAGTAG
pQE30 dNotch EGF23-24 cloning	
Forward	TAGTAGGGATCCATAGAAGGACGATCAGCAGAGGACATAGACGAA TGCTC
Reverse	CTACTAGTCGACCGTCTCGCAGTGCTTGCC
pQE30 dNotch EGF23-25 cloning	
Forward	TAGTAGGGATCCATAGAAGGACGATCAGCAGAGGACATAGACGAA TGCTC
Reverse	CTACTAGTCGACCGTCTGACAATTGATGCCGG
pQE30 dNotch EGF23-24 and EGF23-25 mutagenesis	
dN2325_948_Forward	CATCAATACGGTCGATTGTG
dN2325_948_Reverse	GCGCAATCTCTGCCCTCA
dN2325_986_Forward	GACATCATTGAGTGCTTGAG
dN2325_986_Reverse	CGTCTCGCAGTGCTTGCC
pQE30 hNotch-1 EGF23-24 cloning	

EGF23 Fwd	TAGTAGAGATCTATAGAAGGACGATCAGCAGTCGACATCAACGAGTGCG
EGF24 Rev	TAGTAGCTCGAGCTCACAGAAAGTGCCCCGG
pQE30 hNotch-1 EGF23-24 mutagenesis	
SDM D909V Fwd	GACATCGTCGACTGCCGG
SDM D909V Rev	GGTCTCGCAGTTGCGCCC
pQE30 hNotch-1 EGF23-25 cloning	
EGF23 Fwd	TAGTAGAGATCTATAGAAGGACGATCAGCAGTCGACATCAACGAGTGCG
EGF25 Rev	TAGTAGCTCGAGCTCACAGTGGATCCCGCTG
pQE30 hNotch-1 EGF20-23 cloning	
EGF20EK_F	TAGTAGGGATCCGATGACGATGACAAATCAGCAATCAACAACAACGAGTGTG
EGF23_R	TAGTAGAAGCTTCTATTAGGTCTCGCAGTTGCGCCCACTG
